# Modeling Chemical Looping Gasification with Agroforestry Residues: Validation against Results in a 20 kW_th_ CLG Unit

**DOI:** 10.1021/acs.iecr.5c01725

**Published:** 2025-09-10

**Authors:** Alberto Abad, Luis F. de Diego, María T. Izquierdo, Teresa Mendiara, Francisco García-Labiano

**Affiliations:** 120031 Instituto de Carboquimica (ICB-CSIC), Miguel Luesma Castán, 4, 50018 Zaragoza, Spain

## Abstract

Biomass chemical looping gasification (BCLG) represents an innovative process that allows the generation of non-nitrogen-diluted synthesis gas with low tar content and the potential to avoid CO_2_ emissions. In this work, a 1.5D macroscopic model for the fuel reactor of a BCLG unit was developed and validated to simulate the performance of the system under different operating conditions. The model was developed as simple as possible in order to have a powerful tool to simulate a large number of conditions in a relatively short period of time with low computing effort. However, it has the required complexity to consider the main processes affecting the reaction of the biomass and the oxygen carrier, such as reactor fluid dynamics and the reaction pathway of biomass in the fuel reactor. The main outputs of the model are presented and validated against results from a 20 kW_th_ BCLG unit with two biomasses, namely, pine forest residue and wheat straw pellets. The effects of several operating conditions (temperature, solid circulation rate, solid inventory, and gas flow) on the syngas yield and composition were successfully predicted by the model.

## Graphical abstract

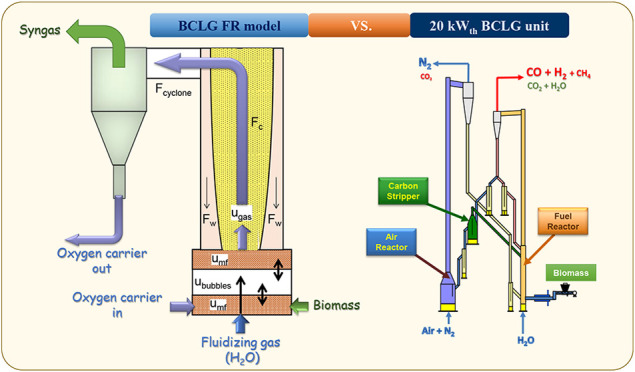

## 1 Introduction

Biomass chemical looping gasification (BCLG) represents an innovative process that allows the generation of non-nitrogen-diluted synthesis gas with low tar content and the potential to avoid CO_2_ emissions.^1^ The BCLG process is based on two interconnected fluidized bed reactors, air and fuel reactors, using a solid oxygen carrier as the bed material circulating between both reactors; see Figure 1. The biomass is fed into the fuel reactor and mixed with a solid oxygen carrier. Steam and CO_2_ are supplied as fluidizing and gasifying agents, respectively. Thus, biomass is devolatized and gasified in the fuel reactor (reactions 1–3) prior to reacting with the oxygen carrier. The oxygen carrier, based on a metal oxide (M_*x*_O_*y*_), provides the oxygen needed for fuel oxidation in a nitrogen-free atmosphere via a redox reaction to Me_*x*_O_*y*–1_ while circulating between both reactors. Thus, gaseous products are oxidized, fully or partially, by the oxygen carrier; see reactions 4 and 5. Note that it is considered that CO and H_2_O are primary oxidized compounds when hydrocarbons react with the oxygen carrier.^2^ The spent oxygen carrier (M_*x*_O_*y*–1_) is subsequently transported to the air reactor to be regenerated with the oxygen from air (reaction 6), closing the loop when returning to the fuel reactor. Partial oxidation of the biomass supports the BCLG process under autothermal conditions because the oxygen carrier also transfers sensible heat to the fuel reactor from the exothermic oxidation reaction in the air reactor.^3^ At the same time, some CO_2_ is produced in the fuel reactor and will be present in the syngas product. This CO_2_ may be separated from the main syngas compounds (H_2_ and CO) by using commercial precombustion processes.^4^

R1 $$biomass\overset{\Delta }{\to }volatiles\;\left(CO,H_{2},CH_{4},...\right)+char\;\left(C\right)$$

R2 $$char\;\left(C\right)+H_{2}O\overset{\;}{\to }CO+H_{2}+ash$$

R3 $$char\;\left(C\right)+CO_{2}\overset{\;}{\to }2CO+ash$$

R4 $$\mathrm{CO}/H_{2},...+nM_{x}O_{y}\overset{\;}{\to }CO_{2}/H_{2}O+nM_{x}O_{y-1}$$

R5 $$C_{n}H_{m},...+\left(n+\frac{m}{2}\right)M_{x}O_{y}\overset{\;}{\to }n\mathrm{CO}+{\frac{m}{2}H}_{2}O+\left(n+\frac{m}{2}\right)M_{x}O_{y-1}$$

R6 $$2M_{x}O_{y-1}+O_{2}\overset{\;}{\to }2M_{x}O_{y}$$

**Figure 1:**
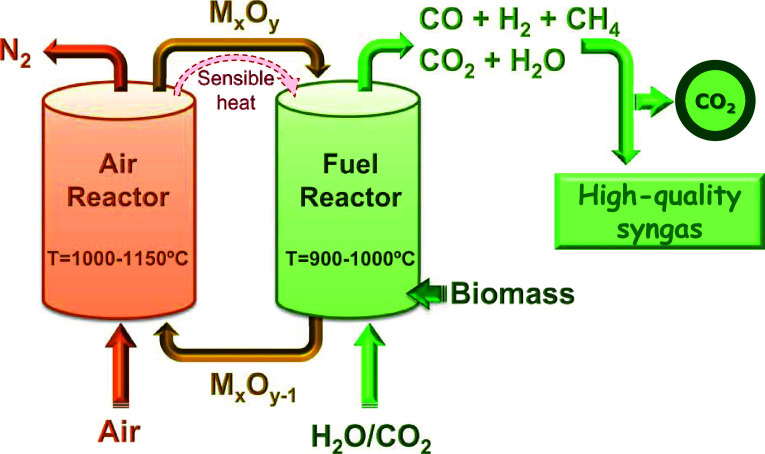
Layout of the BCLG process.

A fundamental part of the reliability of BCLG is based on the behavior of the fuel reactor. The performance of the fuel reactor determines the char and hydrocarbon conversion into CO and H_2_, which mainly affects the syngas yield and composition in the CLG process. Operational conditions should be selected in order to maximize the char conversion and CO and H_2_ production. For that, experimental results suggest that the temperature in the fuel reactor should be in the 900–1000 °C interval.^5^ This fact implies that the required temperature in the air reactor would be about 1000–1150 °C.^3^ Also, both fuel and air reactors are considered adiabatic reactors, and the oxygen/fuel ratio in the fuel reactor should be optimized to maximize the syngas yield. The syngas yield increases as the oxygen-to-fuel ratio decreases,^5^ but this parameter should be high enough to support the autothermal operation of the CLG unit.^3^

Modeling the fuel reactor would be helpful for designing, optimizing, and scaleing up the process in order to obtain high biomass conversion in a BCLG system. Models often applied to chemical looping processes can be included in two general groups:^6^ (1) macroscopic models based on empirical correlations for the fluid dynamics of a fluidized bed; and (2) multiphase CFD-based models. Available results from macroscopic or CFD models to simulate the performance of a CLG unit are scarce.

Understanding the processes and the behavior of fuel conversion in the biomass chemical looping gasification (BCLG) technology is key to designing the fuel reactor and optimizing the operating conditions that allow maximizing synthesis gas yield and minimizing CO_2_ emissions into the atmosphere. This task can be arduous and difficult to achieve by using highly detailed models. Thus, computing fluid dynamics (CFD) models have been mostly applied to simpler reactors, e.g., to understand the oxygen carrier relevance on biomass gasification in a batch-fashion reactor.^7–9^ On the contrary, macroscopic models have been used to evaluate the effect of several operating conditions in biomass gasification, such as temperature and steam-to-biomass ratio.^10^ However, these models need to be applied to the existing environment of a chemical looping unit, and they should be validated against experimental results achieved during the operation of a BCLG unit. Interestingly, Graf et al.^11^ developed a CFD model for the BCLG process, which was developed for a 1 MW_th_ unit at Darmstadt University of Technology. They applied the model to a few experimental conditions, as they highlight the high computational demand of these methods. For a good prediction of the experimental results, they concluded that the devolatilization and gasification steps should be properly described.

In this work, a macroscopic model is developed to optimize the BCLG process and make predictions about the fuel reactor behavior over different operating conditions. This model considers the fluid dynamics of the fuel reactor adapted to the design and operating conditions used in the BCLG unit at ICB-CSIC operated at the 20 kW_th_ scale with ilmenite as an oxygen carrier and two biomasses as fuel: pine forest residue (PFR)^5^ and wheat straw pellets (WSPs).^12^ The fluid dynamics model is simultaneously solved with the mass balance equations corresponding to the biomass conversion and oxygen transference from the oxygen carrier, following the reactions described above. The simpler description of the fluid dynamics compared to CFD models allows prediction in multiple operating conditions with low computational demand. Thus, model predictions were compared to several experimental results in the BCLG unit in order to validate the model.

## 2 Model Description

The mathematical model developed focuses on the fuel reactor behavior of a BCLG unit at ICB-CSIC, operated at the 20 kW_th_ scale with ilmenite as the oxygen carrier and wheat straw pellets (WSPs) or pine forest residue (PFR) as fuels.^5,12^ A description of this unit is presented in the Supporting Information. Mainly, it is composed of a fuel reactor, an air reactor, and a carbon stripper. In this work, the fuel reactor is modeled, but conditions in the air reactor and carbon stripper are considered regarding the oxygen carrier conversion at the fuel reactor inlet and the gas flow coming from the carbon stripper, respectively. Main dimensions are presented in Table 1. The carbon stripper was designed to separate unconverted powdered char particles from the oxygen carrier and recirculate them to the fuel reactor to increase the char conversion. However, the carbon stripper was not able to entrain unconverted char from oxygen carrier particles because pelletized biomass was fed. So, char recirculation from the carbon stripper to the fuel reactor was not included in the model, in contrast with previous works with powdered coal.^13,14^

**Table 1:** Main Dimensions of the Fuel Reactor

reactor geometry	symbol	value
height of the bottom part (m)	*H*_bottom_	1.2
height of the upper part (m)	*H*_up_	2.8
diameter of the bottom part (m)	*d*_bottom_	0.102
diameter of the upper part (m)	*d*_up_	0.081
height of the biomass feeding (m)	*H*_fuel_	0.05
height of solids from loop seal (m)	*H*_LS_	0.10
height of the gas from CS (m)	*H*_CS_	1.0
number of nozzles (gas distributor)	*N*_nz_	72

A simplified diagram of the fluid dynamics in the fuel reactor is presented in Figure 2. The model was developed to be as simple as possible in order to have a powerful tool to simulate a high number of conditions in a relatively short period of time with a low computing effort. However, it has the required complexity to consider the main processes affecting the reaction of the biomass and the oxygen carrier, such as reactor fluid dynamics and the reaction pathway of biomass in the fuel reactor.

**Figure 2:**
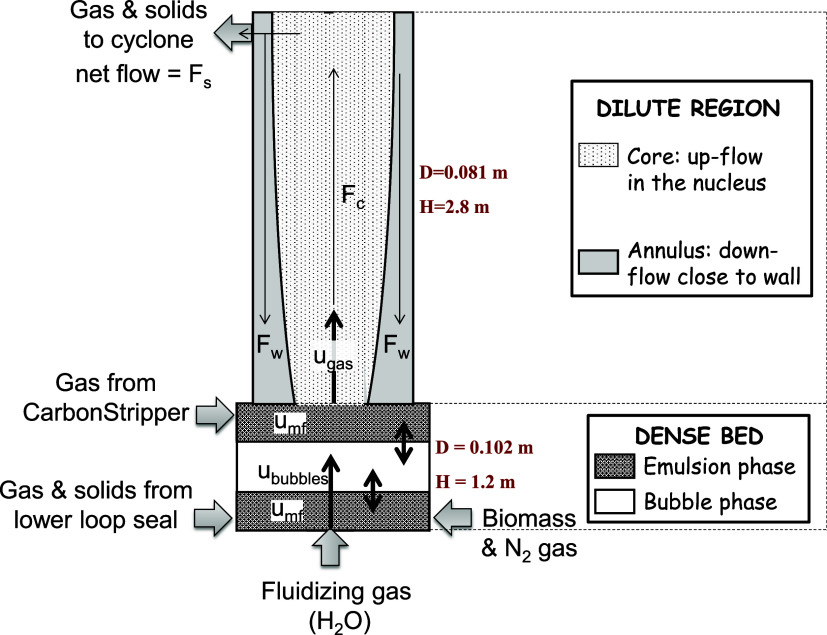
Simplified diagram of gas and solid flows in the fuel reactor of the BCLG unit.

Fluid dynamics is considered to be a 1.5-dimensional macroscopic model based on empirical and semiempirical expressions.^15^ This model has been successfully adapted to simulate the combustion of coal in a chemical looping combustion (CLC) unit^13^ and validated against results in a 100 kW_th_ CLC unit.^14^ Now, it is modified considering the specific conditions for biomass gasification in the 20 kW_th_ BCLG unit.^5,12^ Thus, the fuel reactor is a fluidized bed working at the slugging regime in the bottom bed, with the main gas flow coming from the distributor plate. Minor gas flows come from the lower loop seal and biomass feeding. Biomass is physically mixed with the oxygen carrier in the bottom part of the fuel reactor, where it is pyrolyzed and gasified. The oxygen demanded for the partial oxidation of these gaseous compounds is supplied by the partially oxidized oxygen carrier circulated from the air reactor, which enters the lower loop seal. In the upper part, the high-velocity regime is achieved due to its smaller diameter and the addition of gas coming from the carbon stripper. Solid particles, namely, oxygen carrier or unconverted char, are not entrained from the carbon stripper to the fuel reactor. The stream of solids exiting from the fuel reactor to the cyclone is composed of the gas products from biomass conversion, the reduced solid oxygen carrier, and unconverted char from biomass. Main tools required for the model development have been previously presented, namely, (1) main dimensions and operating conditions of the 20 kW_th_ BCLG unit presented in Tables 1 and 2;^5,12^ (2) properties and reduction kinetics of ilmenite particles;^16^ and (3) main properties and gasification kinetics of WSP and PFR.^17^ A detailed description of the model is found in the Supporting Information.

**Table 2:** Operating Conditions for the Selected Tests to Validate the Fuel Reactor Model^5,12^

								gas flows (m^3^/h, STP)			
biomass test	power, *P*_t_ (kW)	fuel feeding rate, *F*_fuel_ (kg/h)	temperature in FR, *T*_FR_ (°C)	pressure drop, Δ*P*_0_ (kPa)	solids circulation rate, *F*_s_ (kg/h)	oxygen ratio in FR, λ_FR_	carbon conversion in FR, *X*_C,FR_ (%)	H_2_O to FR, *F*_g,in_	N_2_ from fuel feeding, *F*_g,fuel_	N_2_ from LS, *F*_g,LS_	total N_2_ from CS, *F*_g,CS_
WSP-1	18.3	3.84	882	7.9	70	0.30	79.8	2.99	0.50	0.03	4.80
WSP-2	18.3	3.83	897	7.3	43	0.29	91.3	2.49	1.50	0.30	4.25
WSP-3	18.3	3.83	910	9.2	79	0.32	90.3	2.49	1.50	0.30	4.25
WSP-4	19.0	3.98	891	6.5	114	0.23	81.9	2.49	1.50	0.60	6.80
WSP-5	19.0	3.98	986	6.1	445	0.20	82.0	2.49	1.50	0.60	6.80
WSP-6	19.0	3.98	983	6.2	435	0.14	80.5	2.49	1.50	0.60	6.80
WSP-7	19.0	3.98	978	7.7	438	0.14	72.1	2.49	1.50	0.45	6.69
WSP-8	19.0	3.98	879	4.7	100	0.06	73.7	2.49	1.50	0.60	6.60
WSP-9	18.5	3.88	947	5.9	98	0.39	93.3	2.49	1.50	0.70	6.95
WSP-10	18.5	3.88	939	7.3	150	0.44	89.2	2.49	1.50	0.56	6.95
WSP-11	18.5	3.88	934	7.0	101	0.42	91.8	2.49	1.50	0.75	6.97
WSP-12	18.5	3.88	948	10.4	110	0.37	97.6	3.11	1.50	0.90	4.15
WSP-13	18.5	3.88	936	8.0	160	0.37	86.0	4.11	1.50	0.92	4.17
WSP-14	18.5	3.88	928	7.5	160	0.32	97.8	2.49	1.50	0.55	7.43
PFR-1	21.6	4.33	916	9.9	205	0.06	80.5	2.49	1.50	0.93	4.15
PFR-2	21.6	4.33	920	15.7	205	0.44	84.3	2.49	1.50	0.93	7.15
PFR-3	21.6	4.33	935	6.9	124	0.24	78.4	2.49	1.50	0.90	7.31

A detailed description of the material and methods used in the experimental campaign is found elsewhere.^5,12^ Concentrate Norwegian natural occurring ilmenite from Titania AS was used as an oxygen carrier, and it was previously activated in its own unit. This oxygen carrier was formerly proposed by Leion et al.^18^ as an oxygen carrier, and it has been used in several chemical looping units for combustion and gasification of solid fuels.^19^ Pine forest and wheat straw biomasses were previously pelletized for proper use in the BCLG unit. Relevant for the present work is knowing the data selected for model validation. Thus, the gas composition at the FR outlet (CO_2_, CO, H_2_, and CH_4_) was measured online after gas tar cleaning, which was also measured by gas chromatography–mass spectrometry (GC–MS). Also, C2–C5 hydrocarbons were measured via gas chromatography. Because the reaction kinetics of tars and C2–C5 hydrocarbons with the oxygen carrier are not known, as well as these compounds were of low relevance, they were treated as CH_4_ for modeling purposes. The temperature and pressure in the reactor were monitored by using K-type thermocouples and online pressure taps, respectively.

## 3 Results and Discussion

The model has been used to simulate the gasification behavior of two kinds of biomasses, namely, pine forest residue (PFR) and wheat straw pellets (WSPs) with additives, which have been used by ICB-CSIC in a BCLG unit at the 20 kW_th_ unit with ilmenite as the oxygen carrier.^5,12^ The model was adapted to consider the geometry and operating conditions used in this BCLG unit; see Tables 1 and 2. Among the available experimental results, those suitable for model validation were selected. These tests must meet the following conditions:

(I) Test at a steady state for carbon balance: Tests that showed either carbon accumulation in the FR or a higher carbon conversion rate than that in the fuel feeding were disregarded. These conditions were usually found during transition periods after modifications in operating conditions, mainly FR temperature or solids circulation rate.
(II) Test with a suitable oxygen balance in the fuel reactor: The oxygen transferred by the oxygen carrier in the FR should be equal to the oxygen gained in gases. Nevertheless, the oxygen transferred in the AR may be different from the oxygen transferred in the FR. The existence of this condition and its impact on oxygen carrier conversion are evaluated below.

Then, the tests shown in Table 2 were simulated and validated against the experimental results. In addition, a dedicated analysis was performed to evaluate the behavior of the solids flow in the exit zone to the cyclone.

### 3.1 Outputs from the Model

To show the general outputs from the model, simulation results for the WSP-1 and WSP-7 tests (see Table 2) are presented. Test WSP-1 is characterized by a low temperature and low solids circulation rate, whereas test WSP-7 exhibits the opposite conditions. The main outputs of the model are presented as follows: (1) the fluid dynamics structure of the reactor, e.g., profiles of concentration and solids flow in the dilute zone; (2) the axial profiles of gas composition and flows (CO, H_2_, CH_4_, CO_2_, and H_2_O); (3) the conversion of the oxygen carrier and char in the reactor; (4) the char concentration in the reactor; and (5) the gas composition and solids flow at the reactor exit. From these outputs, the syngas yield and the fraction of unconverted char exiting the fuel reactor were calculated. The solids conversion at the fuel reactor inlet was determined by the model to fit the oxygen flow transferred from the oxygen carrier.

The axial profiles of gas and solid concentrations in the fuel reactor for test WSP-1 are shown in Figure 3 and for test WSP-7 are shown in Figure 4. From the profiles of solids concentration, the separation of the dense bed and the dilute region can be easily observed at *H*_b_ = 1.2 m. The dense bed is characterized by a roughly constant solids concentration. Most solids are in the dense bed, and the solids concentration decreases with the reactor height in the dilute region. Both gas velocity and bubble size just above the distributor plate are relatively low. Bubble size quickly increases until it reaches the entire cross section of the reactor, which is characteristic of the slugging regime. Gas velocity increases as the fuel is devolatized and gasified. Both processes happen homogeneously throughout the dense bed. It can be observed that CH_4_ is monotonically accumulated in the dense bed, which is related to the homogeneous devolatilization of pellets in the dense bed and the slow reaction rate of this gas with the oxygen carrier. In this CLG unit, N_2_ coming from the carbon stripper causes an increase in the gas velocity. Often, adding N_2_ doubles the existing gas flow in the fuel reactor. This phenomenon would not happen in an industrial CLG unit without a carbon stripper. Then, the gas velocity highly increased when it passed from the dense bed to the dilute region. This is due to the decrease in cross section through the gas flows, i.e., the entire reactor section in the dense bed and the core section of the dilute region in the riser. Note that the core section is smaller than the reactor section in the riser since a core-annulus structure was assumed, with gas and solids ascending by the core and solids descending by the annulus.

**Figure 3:**
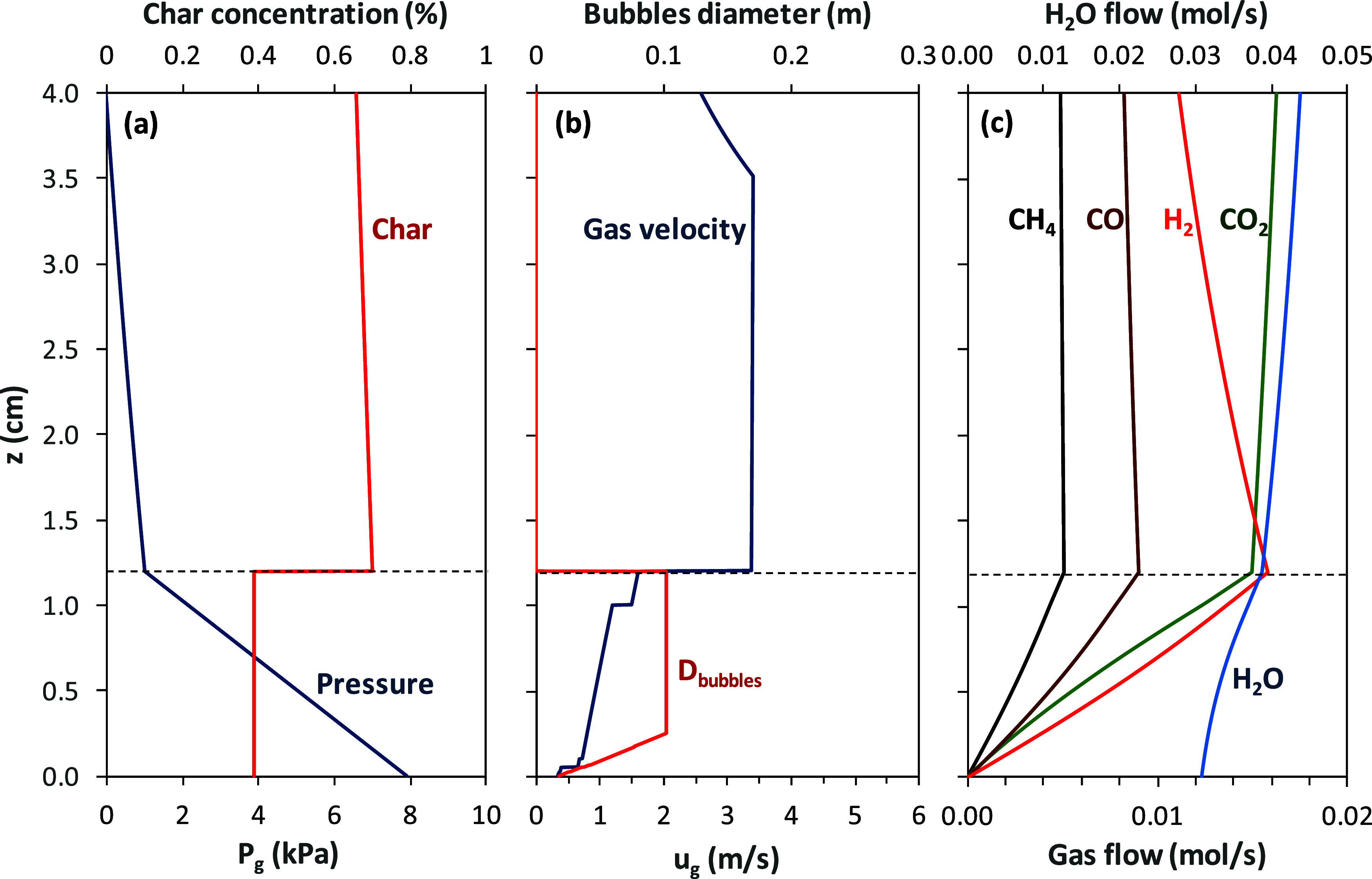
Longitudinal profiles in the fuel reactor of (a) gauge pressure, *P*_g_, and char concentration, *C*_C_; (b) bubble diameter and gas velocity; and (c) flow of gases for test WSP-1.

**Figure 4:**
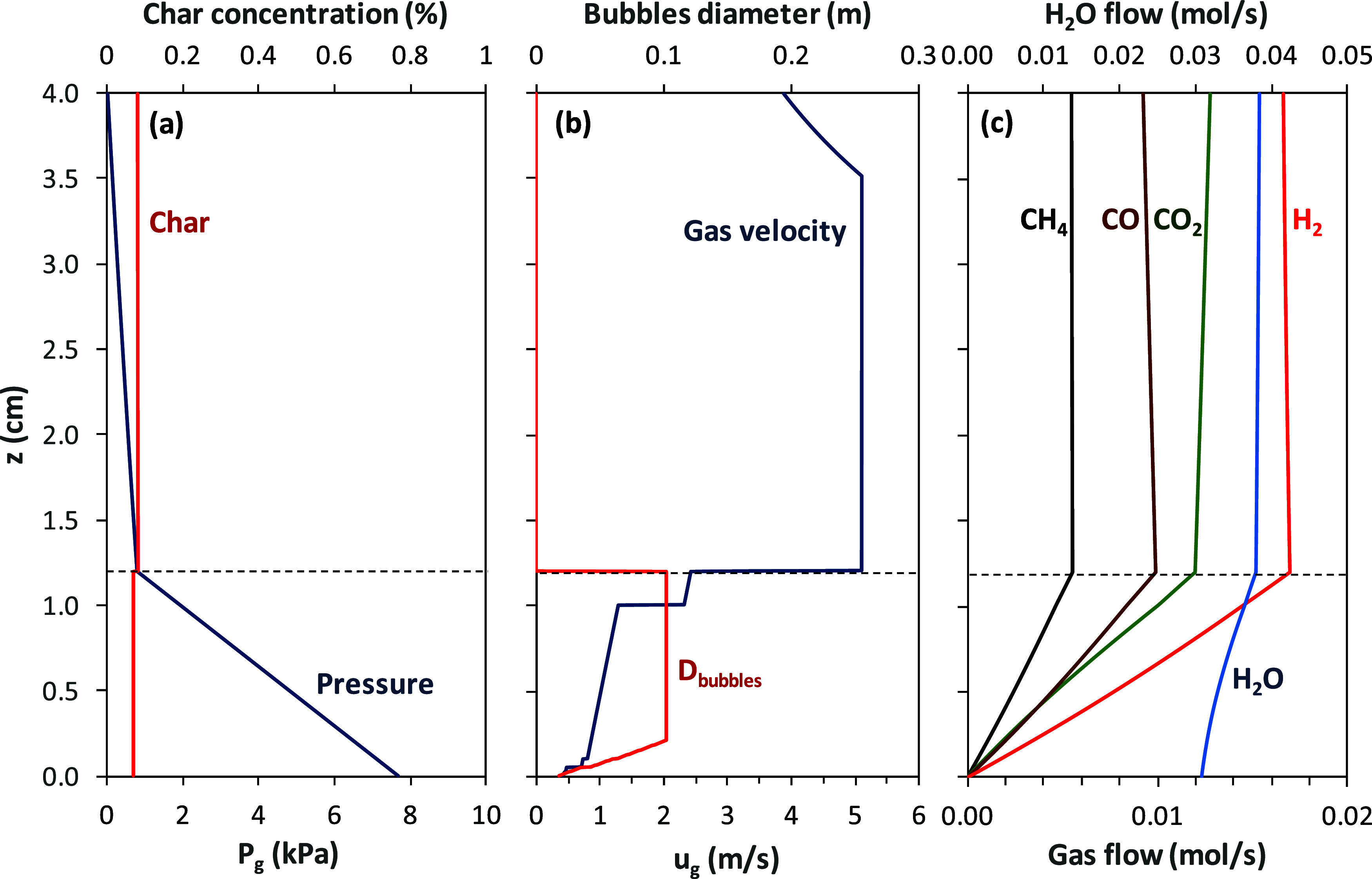
Longitudinal profiles in the fuel reactor of (a) gauge pressure, *P*_g_, and char concentration, *C*_C_; (b) bubble diameter and gas velocity; and (c) flow of gases for test WSP-7.

Relevant differences are observed among the results in tests WSP-1 and WSP-7. Char concentration was higher in test WSP-1 than in test WSP-7 (see Figures 3a and 4b), mainly due to the lower gasification rate at 882 °C in test WSP-1 compared to the faster gasification rate in test WSP-7 at 978 °C. Thus, more char is accumulated in the fuel reactor as the temperature decreases, which was inferred during the experimental campaign. The lower temperature in test WSP-1 hinders char gasification in the upper part, which increases the relative relevance of the oxidation products with the oxygen carrier. As a consequence, H_2_ concentration decreases with the riser height in this test; see Figure 3c. In addition, the oxygen carrier was highly oxidized in the air reactor in test WSP-1. The model predicts values for the oxidation degree of solids at the inlet and outlet of the reactor of *X*_OC,FRin_ = 0.99 and *X*_OC,FRout_ = 0.46. Thus, almost half of their oxygen transport capacity is still available for reaction in the dilute region.

On the contrary, generation and consumption of gasification products by char gasification and reaction with the oxygen carrier, respectively, are balanced at the higher temperature in test WSP-7; see Figure 4c. Thus, the variation in CO and H_2_ flows in the riser is of low relevance in test WSP-7. This effect is also related to oxygen carrier conversion in the fuel reactor. The oxygen carrier was highly reduced in test WSP-7, with predicted values of *X*_OC,FRin_ = 0.066 and *X*_OC,FRout_ = 0.027. So, the oxygen available in the solids for reaction in the dilute region was very scarce.

To better appreciate the behavior of the fuel reactor in the dense bed, the profiles of gases in the emulsion and bubbles and the average concentration in the dense bed are shown in detail in Figures 5 and 6 for tests WSP-1 and WSP-7, respectively. At the bottom of the bed, the gas is mainly composed of H_2_O, which is the fluidization gas. Then, gasification products, H_2_ and CO, are accumulated mainly in the bubbles, while CO_2_ is accumulated mainly in the emulsion as a product of gas oxidation by the oxygen carrier. This means that gasification products generated from char in the emulsion are either converted to CO_2_ and H_2_O by reacting with oxygen carrier particles in the emulsion or are diffusing to bubbles where they are accumulated. CH_4_, as a characteristic compound of volatile matter, also monotonically accumulates mainly in the bubbles, which corresponds to the homogeneous devolatilization of pellets in the dense bed.

**Figure 5:**
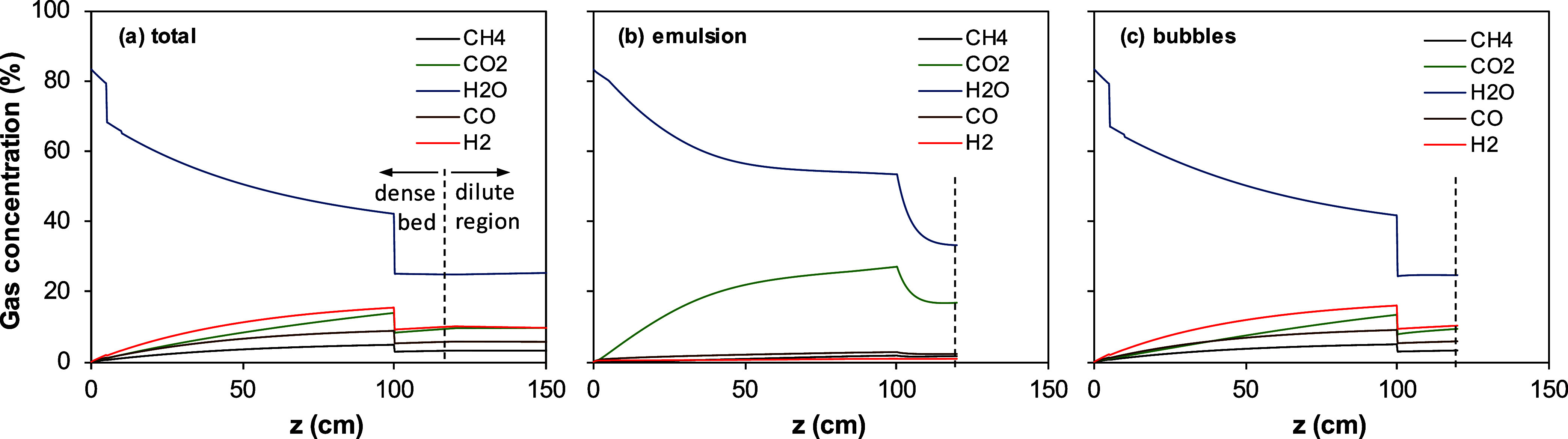
Longitudinal profiles of concentration of gaseous compounds, *C*_g_, in the bottom bed of the fuel reactor for test WSP-1: (a) average concentration, (b) emulsion, and (c) bubbles.

**Figure 6:**
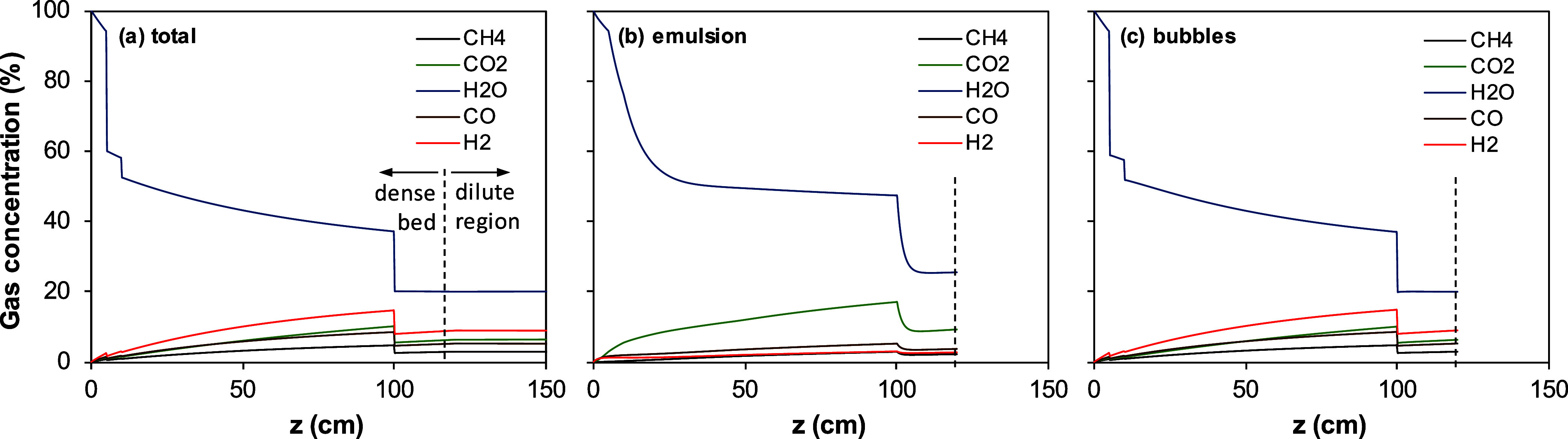
Longitudinal profiles of concentration of gaseous compounds, *C*_g_, in the bottom bed of the fuel reactor for test WSP-7: (a) average concentration, (b) emulsion, and (c) bubbles.

When the top of the dense bed is reached, the gas in the emulsion and bubble is mixed. Because the flow through the bubbles is much higher than that through the emulsion, the gas composition in the dilute region is dominated by the gaseous components in the bubbles. In general, gasification products do not accumulate in the dilute region. This is due to the low solid concentration in this region compared to the dense bed. Due to the lower entrainment velocity of pellets compared to oxygen carrier particles, the concentration of char in the solids is somewhat higher in the dilute region.

The reaction in the dilute region depends on the gas–solid contact efficiency, which is affected by the operating conditions, as described in the Supporting Information. A deeper analysis of this issue was done compared to Figures 5c and 6c for tests WSP-1 and WSP-7, respectively. In both cases, H_2_ and CO flows decrease with the reactor height, while H_2_O and CO_2_ flows increase. This fact suggests that the oxidation of gasification products dominates the generation of H_2_ and CO by char gasification or methane reformation. However, the CH_4_ flow barely changed in any case due to the low reactivity of ilmenite with CH_4_. The decrease in the H_2_ flow was more evident in test WSP-1 than in test WSP-7. This fact was related to the high reactivity with H_2_, the higher solids concentration, and the lower gasification rate at the lower temperature in test WSP-1.

Additional information about the conversion of gases in the dense bed and dilute region comes from a separate analysis of the gasification products (H_2_ + CO), combustion products (CO_2_ + H_2_O), and the total flow (CO_2_ + H_2_O + H_2_ + CO); see Figure 7. In general, the total flow barely changes with the reactor height in the dilute region, indicating that the gasification reaction is of low relevance in this zone. Thus, most gasification products are generated in the dense bed regardless of the char concentration existing in the dilute region. In test WSP-1, there is a clear decrease in the flow of gasification products (CO + H_2_), which is related to the high H_2_ consumption, as mentioned in the discussion of Figure 3c. However, both gasification and combustion reactions were of low relevance in the dilute region for test WSP-7 due to lower oxygen carrier, char concentration, and oxygen availability, as mentioned previously in the discussion of Figure 4c. In addition, the H_2_ + CO flow in test WSP-1 was lower than in test WSP-7 because the oxygen transferred was higher in the former (λ_FR_ = 0.3) than in the latter (λ_FR_ = 0.14).

**Figure 7:**
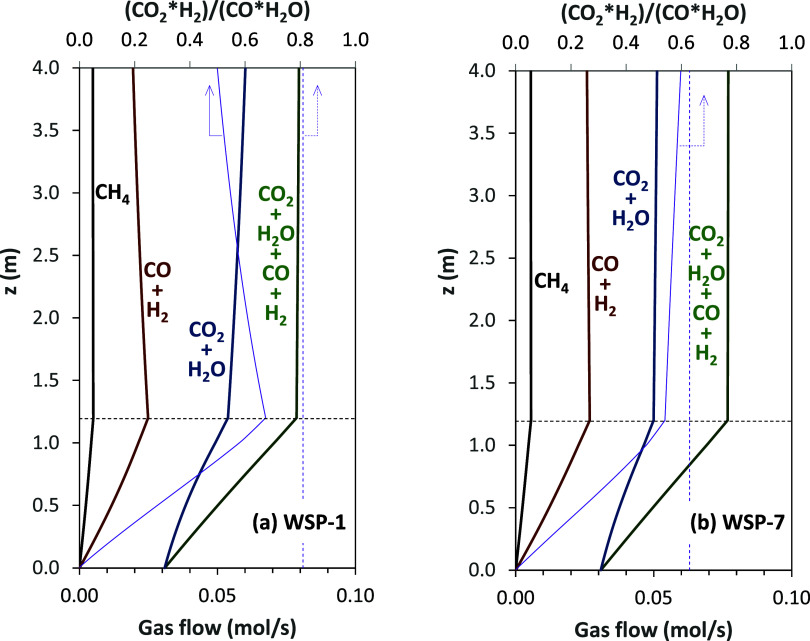
Longitudinal profiles in the fuel reactor of the flows of gaseous compounds and relation of the involved compounds in the water–gas shift (WGS) equilibrium for (a) test WSP-1 and (b) test WSP-7. Broken lines: equilibrium constant for the WGS reaction.

Eventually, the water–gas shift (WGS) reaction kinetics modulates the variations in H_2_, CO, H_2_O, and CO_2_ flows.^20^ The relevance of the WGS reaction is evaluated considering the relation in the gas concentration represented by the equilibrium constant in eq 7.

1 $$K_{\mathrm{eq},\mathrm{WGS}}={\left[\frac{P_{CO_{2}}P_{H_{2}}}{P_{\mathrm{CO}}P_{H_{2}O}}\right]}_{\mathrm{eq}}$$

Figure 7a shows that the relation between the gases involved in the WGS equilibrium (namely, H_2_O, CO, H_2_, and CO_2_) moves away from the equilibrium as the gas rises through the riser in test WSP-1 at low temperatures. This is because the rate of disappearance of H_2_ is higher than that of CO, and the correction is by the WGS reaction. However, this relation is approaching the value at the equilibrium conditions in test WSP-7, indicating that the WGS reaction is relevant at higher temperatures; see Figure 7b.

### 3.2 Validation of the Fuel Reactor Model against Results in the 20 kW_th_ BCLG Unit

Once the processes happening in the different regions of the fuel reactor were described and the results that the model can provide were known, the validation of the model is addressed. One of the most relevant pieces of information given by the model is the flow and concentration of different gaseous compounds at the fuel reactor outlet. Thus, concentrations of CO_2_, CO, H_2_, H_2_O, and CH_4_ can be compared to those measured during the experimental campaign; see Figure 8. The tendency of the gas concentration values to increase was adequately predicted. This fact suggests that the devolatilization process and gasification reaction were properly described in the model. Note that, during a preliminary evaluation, it was found that the product gas composition was highly sensitive to the distribution of volatiles. To evaluate the goodness of predictions, Figure 9a shows a comparison between the experimental values and the predicted ones for the concentration of gases. Most predictions show deviations lower than ±10%. However, in some cases, higher differences between predicted and experimental values can be seen. To better evaluate the observed deviations, Figure 9b shows the individual deviation for each gas concentration in every experimental test. The major deviation is found for the predicted CO concentration. The average deviation and the root-mean-square error (RMSE) are given in Table 3. In general, the concentrations of CH_4_, CO_2_, and H_2_ remain underpredicted, while those of H_2_O and CO remain overpredicted. This fact may be mainly related to modifications of the water–gas shift equilibrium downstream of the fuel reactor, as discussed below. Also, some CH_4_ could be formed by methanation, which is not considered in this work due to a lack of reaction kinetics, but it would be of lower relevance.

**Figure 8:**
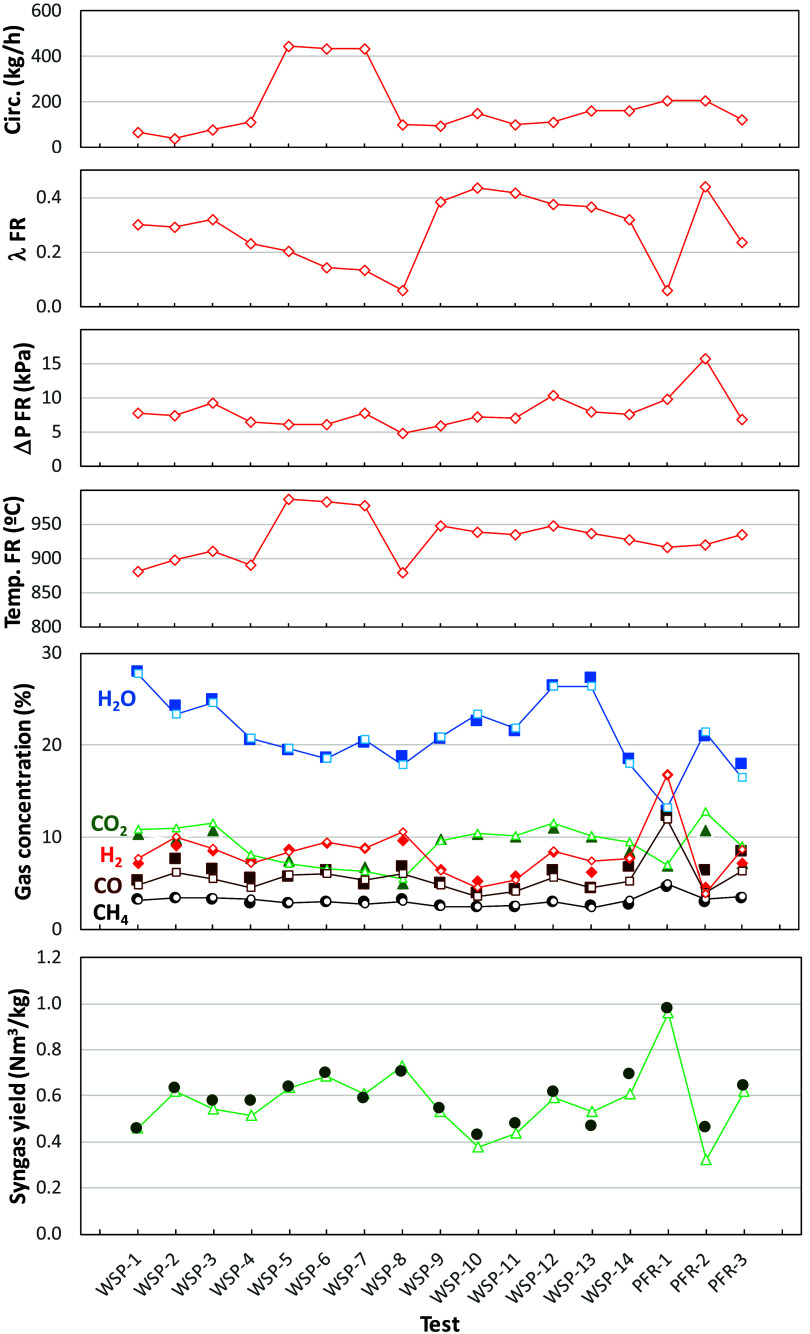
Concentrations of CO_2_, CO, H_2_, H_2_O, and CH_4_ at the fuel reactor exit and syngas yield for tests performed at different operating conditions in the 20 kW_th_ BCLG unit; see Table 2. Open symbols, experimental results; closed symbols, model predictions.

**Figure 9:**
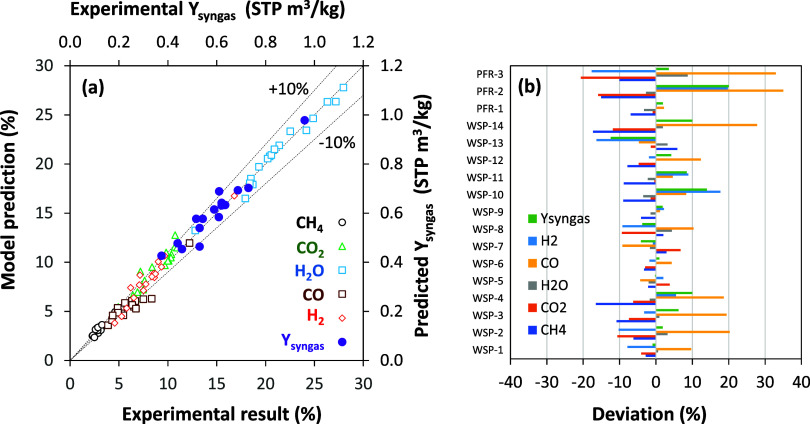
Evaluation of the deviation in the predicted concentration of gases and syngas yield, *Y*_syngas_, by means of (a) comparison between experimental and predicted parameters and (b) individual deviations in each parameter for every experimental test.

**Table 3:** Average Deviation and RMSE for the Predicted Gas Concentrations and Syngas Yields

	average deviation (%)	RMSE
CH_4_	–6.5	9.11 × 10^–2^
CO_2_	–5.1	8.49 × 10^–2^
H_2_O	+0.3	3.15 × 10^–2^
CO	+8.1	1.68 × 10^–1^
H_2_	–0.7	9.92 × 10^–2^
*Y*_syngas_	+3.6	8.17 × 10^–2^

CH_4_ was present at the fuel reactor outlet, but its concentration was lower than those for other gases. CH_4_ is a characteristic compound from volatile matter, and its conversion is rather low (typically 10–20%) due to the low reactivity of ilmenite with this gas; see Figure 10. The low CH_4_ conversion is in line with the relevant CH_4_ content in the gas product when ilmenite has been used in several chemical looping units for combustion or gasification of solid fuels.^19^ It is remarkable that the extremely low conversion predicted in some tests, especially when the oxygen carrier was highly reduced and the solid circulation rate was low.

**Figure 10:**
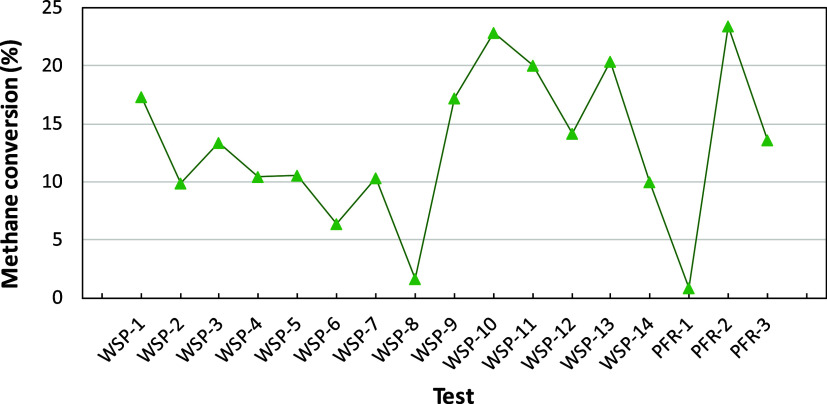
Methane from volatile matter converted to the fuel reactor.

In general, the H_2_ concentration was higher than the CO concentration. This is related to the high molar content of H_2_ in volatiles^21,22^ but also to the high fraction of water in the gaseous products. In this sense, an evaluation of the water–gas shift (WGS) equilibrium was done. Figure 11 shows that, in most cases, the value of the right side of eq 7 from the current composition in each test is higher than the equilibrium constant value, *K*_eq,WGS_. In fact, under equilibrium conditions, there should be more H_2_ and CO_2_ at the expense of less CO and H_2_O. In addition, the temperature at which the gas composition fulfills the equilibrium condition is higher than the current temperature in the fuel reactor in most cases; see the Δ*T* values higher than 0 in Figure 11. Therefore, the gas composition cannot be justified by a modification of the gas composition by the WGS reaction while the gas is cooling downstream the fuel reactor. Therefore, the WGS equilibrium is not achieved in the fuel reactor. These results suggest that the introduction of reaction kinetics for the WGS reaction is necessary to predict the gas composition at the fuel reactor exit and that it cannot be assumed that the WGS equilibrium is fulfilled inside the fuel reactor.

**Figure 11:**
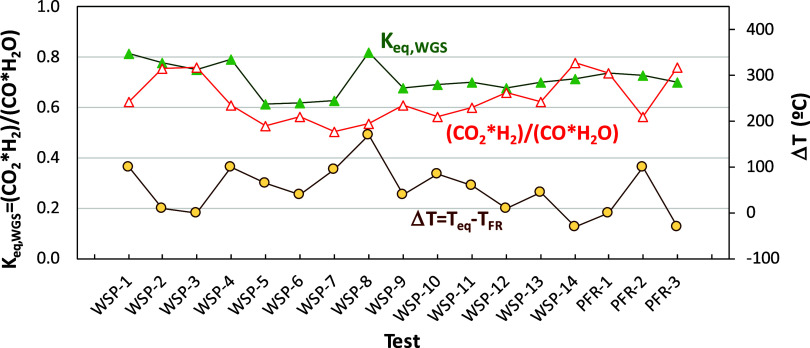
Comparison of the equilibrium constant for the WGS reaction with the value of the right-side term in eq 7 using the current composition at the fuel reactor exit. The incremental temperature, Δ*T*, in the gas to achieve equilibrium conditions is also shown.

Syngas yield is a relevant parameter in CLG in order to evaluate the conversion efficiency of biomass to CO and H_2_. It is defined as the volume of H_2_ and CO produced (Nm^3^) per kilogram of biomass. In general, Figures 8 and 9 show good agreement between the theoretical values predicted by the model and the experimental results obtained from the CLG unit. The estimated error was lower than 10% in most cases, with the average error being +3.6%; see Table 3.

The model is able to calculate the solids conversion at the inlet and outlet of the fuel reactor in order to fulfill the oxygen balance in this reactor, which is defined by the oxygen-to-fuel ratio in the fuel reactor, λ_FR_. The predicted values of the oxidation degree of solids are shown in Figure 12, which are in agreement with experimental results at either the inlet or outlet of the reactor. Note that the oxidation degree of the oxygen carrier was only measured in a few experimental tests, i.e., when solids were extracted and carefully handled to avoid reoxidation with ambient air. The difference between these values at the inlet and the outlet is directly related to the solids circulation rate. Thus, the difference in solids conversion decreases as the solids circulation rate increases.

**Figure 12:**
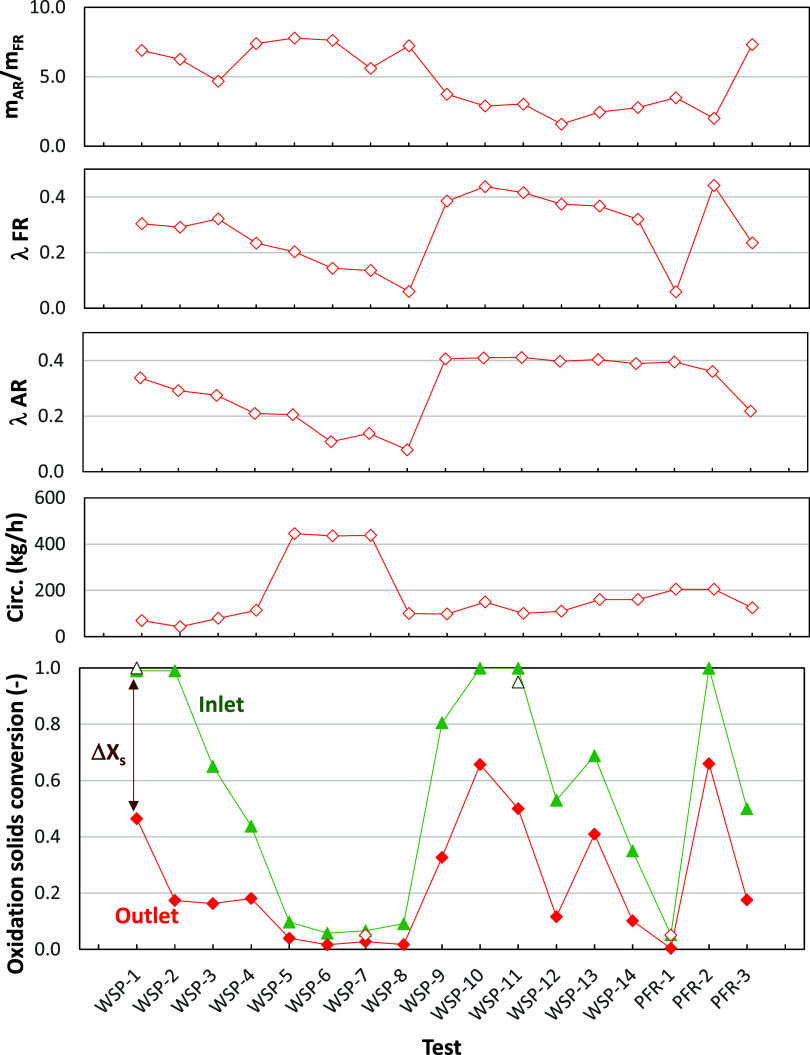
Oxidation degree of oxygen carrier particles at the inlet and outlet of the fuel reactor for tests in Table 2, which were performed in the 20 kW_th_ BCLG unit at several solids inventories in the fuel and air reactor (*m*_FR_ and *m*_AR_), the oxygen-to-fuel ratio transferred in the fuel (λ_FR_) and air (λ_AR_) reactors, and the solids circulation rate. Open symbols, experimental results; closed symbols, model predictions.

A first evaluation of results in Figure 12 shows that the oxygen carrier was initially highly oxidized, which corresponds to the complete oxidation of ilmenite during the thermal pretreatment before it was loaded in the BCLG unit. Then, the oxidation degree of the particles was gradually decreased as it was operated under BCLG conditions. This means that some oxygen transferred to the fuel in the fuel reactor could be taken from the oxygen initially present in the ilmenite particles, in addition to the oxygen supplied by air. Eventually, in tests WSP-5–WSP-8, the oxygen carrier was highly reduced and the oxygen transferred in the air reactor was similar to the oxygen transferred in the fuel reactor. This condition is required to achieve steady-state operation of the CLG unit.

However, there are some tests where the oxidation degree of particles increased again, e.g., tests WSP-9, WSP-10, or PFR-2. Results presented by Abad et al.^23^ show that there is a relation between the operating conditions in fuel and air reactors and the oxidation degree of the solids. For example, a high inventory/reaction rate in the fuel reactor compared to that in the air reactor promotes the oxygen carrier to be highly reduced. However, if the air excess or solid inventory in the air reactor is oversized, the solids are highly oxidized. This fact was confirmed by Cabello et al.^24^ during chemical looping combustion (CLC) conditions by comparing results achieved with either a high or a low excess of air, with the oxygen carrier being highly or scarcely oxidized, respectively. In the absence of air, similar to what happens in CLG, these authors also showed low oxygen carrier conversion values during the operation of chemical looping reforming (CLR) of methane.^25^ The CLG process operates with an oxygen deficit in the air reactor compared to the amount required for full fuel consumption. Thus, usually the oxygen carrier is highly reduced in the BCLG process.^26^

A deeper analysis can be done considering the effect of the oxygen being transferred in the air reactor, λ_AR_, on the estimated degree of oxidation of solids at the fuel reactor exit; see Figure 13. First, conditions at steady state are discussed. These tests are characterized by an agreement in the oxygen balance both in the air and fuel reactors. Thus, the oxygen flux transferred from air to the oxygen carrier in the air reactor should be equal to the oxygen flux from the oxygen carrier to the fuel in the fuel reactor. To evaluate the oxygen balance, the oxygen index, Φ, is defined as the ratio between the oxygen-to-fuel ratio in the fuel and air reactors; see eq 8.

2 $$\Phi =\frac{\lambda_{\mathrm{FR}}}{\lambda_{\mathrm{AR}}}$$

**Figure 13:**
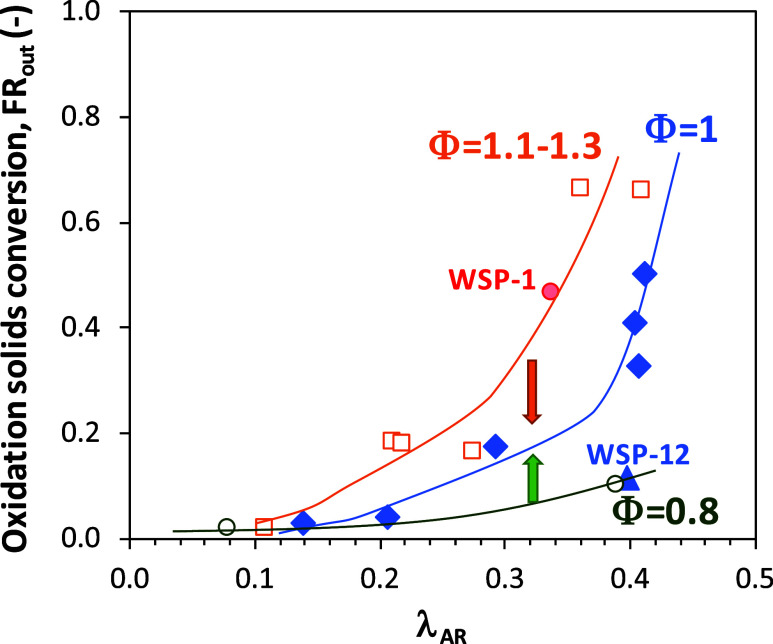
Oxidation degree of oxygen carrier particles at the fuel reactor exit as a function of the oxygen-to-fuel ratio transferred in the air reactor (λ_AR_) for different ratios between the oxygen transferred in the fuel and air reactors, Φ = λ_FR_/λ_AR_.

At the steady state, Φ = 1, the oxidation degree of solids exiting the fuel reactor increases as the oxygen-to-fuel ratio in the air reactor, λ_AR_, increases. In most cases, the oxygen being transferred in the fuel reactor is lower than the oxygen available in the oxygen carrier because the solid inventory in this reactor was not enough to completely deplete the oxygen in the solids. This fact was relevant due to the low reactivity of ilmenite with the reacting gases. However, ilmenite oxidation was fast enough to deplete all of the available oxygen in the air reactor. As a consequence, the oxygen carrier was more oxidized, as more oxidizing conditions were used in the air reactor. Most of these tests were performed with a solids inventory in the fuel reactor of 5–6 kg. One test (see test WSP-12 in Figure 13) was performed with a higher solids inventory in the fuel reactor (8.6 kg). In this case, the reduction degree of the oxygen carrier was promoted in the fuel reactor; i.e., the oxidation degree of solids at the fuel reactor exit decreased. Also note that solids in test WSP-1 presented a relatively high oxidation degree. This fact was affected by the high degree of oxidation of particles at the beginning of the BCLG tests.

Second, tests under transitory periods in the BCLG unit are evaluated, but the steady state is for the fuel reactor conditions. When Φ > 1, the general trend with λ_AR_ was similar to tests at the steady state. However, the estimated oxidation degree was higher than those predicted for tests at Φ = 1. In these cases, the oxygen transferred in the fuel reactor was higher than the oxygen transferred in the air reactor. This means that some oxygen transferred in the fuel reactor is taken from the oxygen initially available in the solids. As a consequence, the oxidation degree of particles will decrease with time until reaching a steady state in the BCLG unit; see Figure 13. A contrary result was observed in some tests at Φ < 1.

### 3.3 Evaluation of the Solids Entrainment Rate to Cyclone

A key parameter for the performance of the fuel reactor is the evaluation of solids exiting the fuel reactor. The entrained solids from the dense bed flow through the core in the dilute region. When they achieve the exit zone, a fraction of solids follows the gas stream toward the cyclone, but another fraction of solids gets separated from this stream and takes a downward direction close to the reactor wall, as described in Figure 2. In addition, coarse char particles are also entrained, aided by the smaller ilmenite particles, even though the terminal velocity for an isolated devolatilized pellet may exceed 10 m/s.^27^ The fraction of solids exiting the fuel reactor is characterized by the backflow ratio, *k*_b_, or the entrainment probability, *p*_ent_, as described by eq 9. However, this value is usually unknown, and it depends on the exit geometry^28–30^ and gas and solids flows.^15^ The upward flow of solids by the core could not be experimentally determined in the CLG unit, but it was calculated by the model, for both the oxygen carrier and char particles. Therefore, the entrainment probability, *p*_ent_, was calculated by using this value in eq 9 and the solids circulation rate determined experimentally; see Table 2.

3 $$p_{\mathrm{ent}}=\frac{1}{k_{b}+1}=\frac{F_{s}}{F_{c,H_{r}}}$$

Figure 14 shows that the entrainment probability depends on the slip velocity, *u*_slip_, i.e., the difference between the gas velocity and the terminal velocity of the particles. In addition, the entrainment probability was slightly higher for oxygen carrier particles than for char particles because the terminal velocity of the oxygen carrier (*u*_t,OC_ = 1.6 m/s)^31^ was higher than that for pellets in the solids mixture (*u*_t,pellet_ = 1.9 m/s).^27^ The values calculated here are specific to this 20 kW_th_ BCLG unit at ICB-CSIC, and in general, they are lower than those determined for industrial facilities with lower solids flux values.^15^ For example, in a 12 MW CFB boiler, the entrainment probability was between 0.3 and 0.4 when the slip velocity varied from 1.5 to 2.5 m/s but with a solids flux (∼15 kg m^–2^s^–1^) lower than those found in the BCLG unit (50–80 kg m^–2^s^–1^). However, the fuel reactor in a chemical looping unit has relevant differences compared to most studied circulating fluidized bed boilers, listed as follows:

- The oxygen carrier particles have different particle sizes and densities than common materials used in fluidized beds. Usually, oxygen carrier particles are denser, which may cause a lower solids entrainment rate or probability.
- The solids circulation rate in chemical looping may be limited by the solids flow from the air reactor. Anyway, the solids circulation rate will be in a dynamic equilibrium with the solids inventory in the reactor. Namely, the model is able to predict the solids circulation rate for a given solids inventory or *vice versa*. This relation was predicted adequately.
- The exit geometry highly affects the entrainment probability. Therefore, it would be quite difficult to achieve a reliable comparison between mass flows achieved in the BCLG unit and in common circulating fluidized bed boilers.

**Figure 14:**
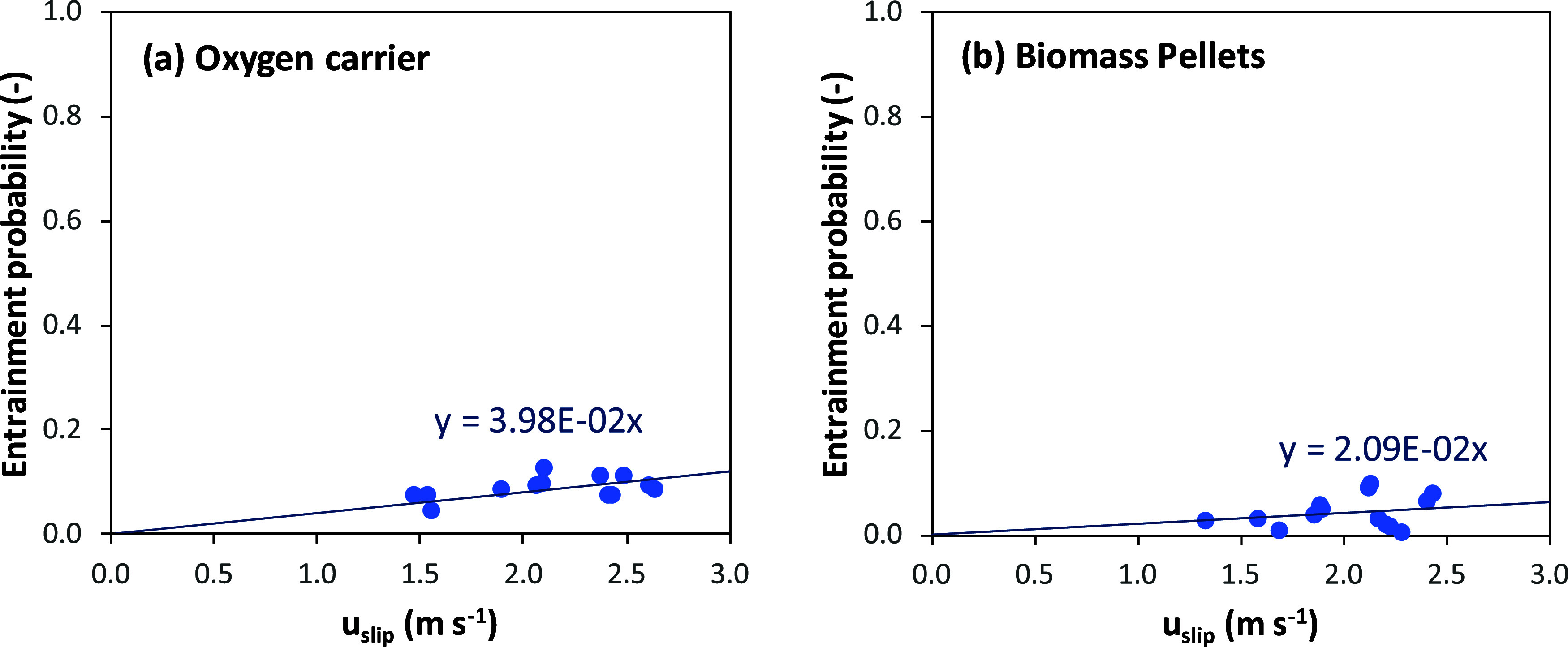
Entrainment probability of solids: (a) oxygen carrier and (b) biomass pellets in the exit zone of the fuel reactor as a function of the slip gas velocity.

In addition, to the best of our knowledge, no information is available about the entrainment probability of coarse particles (devolatilized pellets in our case) in a flow of smaller particles (ilmenite in our case).

The entrainment probability is a key parameter to predict the residence time of solids in the fuel reactor and, thereby, the char conversion. It should be determined for each reactor, as it is a function of the exit geometry, gas velocity, and solids flux. The use of entrainment probability determined for the BCLG unit at ICB-CSIC allows simulation of the behavior of this unit as a function of any operating condition. In this way, the model is able to predict the gas composition at the fuel reactor exit as a function of the oxygen carrier conversion at the fuel reactor inlet. This parameter will define the oxygen:fuel ratio in the fuel reactor. For the simulation of an industrial BCLG unit, the entrainment probability should be estimated, and then, the gas composition could be predicted by the model. Thus, these results have been used for a preliminary simulation of the behavior of a 200 MW_th_ BCLG unit,^32^ which will be completed shortly. Note that the model developed in this work was done by including the specific fluid dynamics of the 20 kW_th_ BCLC unit, e.g., by including the low gas velocity in the bottom part or the addition of gas from the carbon stripper. In order to extrapolate these results to larger scales, the corresponding fluid dynamics of the scaled-up fuel reactor should be used, which generally can be done by considering the information compiled by Pallarès and Johnsson.^15^ In order to predict the expected results on larger scales, the fluid dynamics part of the model should be correspondingly modified. However, the general reaction scheme considering the kinetics for the oxygen carrier and biomass particles could be maintained as described in this work. Therefore, the validated model will be a helpful tool for designing, optimizing, and scaling up the BCLG process in order to identify operating conditions and basic design parameters to achieve both high biomass conversion and high CO_2_ capture rates.

## 4 Conclusions

A model to describe the behavior of the fuel reactor of a biomass chemical looping gasification (BCLG) unit was developed. For validation purposes, the model considered the design parameters and experimental conditions existing in a 20 kW_th_ BCLG unit at ICB-CSIC. Ilmenite was the oxygen carrier, and WSP or PFR was the biomass fuel. The evaluation of the main outputs of the model was useful to understand the chemical processes happening in the fuel reactor, while biomass was converted and partially oxidized by reacting with oxygen carrier particles. Thus, char gasification mainly happened in the dense bed, while a relevant fraction of the oxygen transferred happened in the dilute region. The syngas was enriched in H_2_ and CO as more char was gasified and less oxygen was consumed from the oxygen carrier. The product gas does not achieve the water–gas shift equilibrium at the evaluated temperatures. In addition, CH_4_ conversion was relatively low due to the low reactivity of ilmenite with this gas.

The concentrations of gases (CO_2_, CO, H_2_, H_2_O, and CH_4_) at the fuel reactor exit predicted by the model were compared with the experimental values. In general, good agreement was found, and the general tendency of the gas concentrations was adequately predicted. The syngas yield in the fuel reactor was also properly predicted in all cases. Gas velocity and solids flow affected the entrainment probability, which was lower for char than for the oxygen carrier. This parameter should be determined for a proper simulation of the BCLG process.

## Supplementary Material



## Notes

Detailed description of the fuel reactor model, including reactor geometry, fluid dynamics, and mass balances. (PDF)
