# Supplementary material for: Modeling Chemical Looping Gasification with Agroforestry Residues: Validation against Results in a 20 kWth CLG Unit
Source: Ind Eng Chem Res. 2025 Sep 10;64(38):18576–89. doi: 10.1021/acs.iecr.5c01725 (PMC12464985; doi:10.1021/acs.iecr.5c01725)
Supplement: Supplementary file 1 [file ie5c01725_si_001.pdf]

SUPPORTING INFORMATION

Modelling chemical looping gasification with agroforestry residues: validation against results in a 20 kW_{th} CLG unit

Alberto Abad, Luis F. de Diego, María T. Izquierdo, Teresa Mendiara, Francisco García-Labiano*

Instituto de Carboquimica (ICB-CSIC), Miguel Luesma Castán, 4, 50018 Zaragoza, Spain

**Corresponding Author: abad@icb.csic.es*

1. MODEL DESCRIPTION

The mathematical model developed is focused on the fuel reactor behaviour of a 20 kW_{th} Biomass Chemical Looping Gasification (BCLG) unit at ICB-CSIC, which has been operated with ilmenite being the oxygen carrier and wheat straw pellets (WSP) or pine forest residue (PFR) being the fuels.[S1,S2] The model includes the main processes affecting to the reaction of the biomass and the oxygen carrier, such as reactor fluid dynamics and the reaction pathway of biomass in the fuel reactor.

1.1. Main assumptions and inputs for the model development. The main inputs of the model are shown in Tables S1-S4. They can be divided into the following categories:

a) Design of the reactor: the size of the reactor (diameter, height), as well as the inlet height of the biomass and gas from the carbon stripper is required by the model. The reactor geometry at the solids exit also affects to the fraction of solids flowing by the core, F_c, with the circulating solids being entrained to the cyclone, F_s. This characteristic of the reactor is given by the backflow ratio, k_b, or the entrainment probability, p_{ent}. These parameters will be determined by the model.
b) Operational conditions: in this category the gas flow and composition introduced into the reactor, the temperature and pressure of the reactor, the pressure drop in the whole fuel reactor, the biomass feeding rate, the solids circulation rate from the air reactor, and the oxygen transferred from the oxygen carrier are described. The pressure drop into the reactor gives the total amount of solids inside the reactor. The oxygen being transferred in the fuel reactor is defined by the oxygen to fuel ratio in the fuel reactor, λ_{FR}, and it determines the calculated oxidation degree of the oxygen carrier at the fuel reactor inlet.
c) Solids properties, both oxygen carrier and biomass: physical and chemical properties of solids are included in this category. On the one hand, physical properties are important to know the fluid dynamics of the reactor. In this sense, it is necessary to know the size, density and shape of the solid particles. On the other hand, the chemical properties make reference to composition of solids as well as the reactivity of the oxygen carrier with the reacting gases (CO, H_2 and CH_4), besides the reactivity of biomass char gasification with H_2O and CO_2. Pelletized biomass is fed, both for WSP and PFR. They maintain its pelletized shape after devolatilization and gasification, but some reduction in their dimension was observed.

Description of the BCLG unit and operating conditions of performed tests were included in previous works.[S1,S2] A scheme of the BCLG unit is shown in Figure S1 and the main dimensions of the fuel reactor are in Table S1. This unit consists of a fuel reactor, an air reactor and a carbon stripper. The fuel reactor is fluidized by steam and biomass is fed at the bottom part of the fuel

reactor by means of a screw feeder, and helped by a N_2 flow. Also, the oxygen carrier is introduced at the bottom part, but coming from the lower loop seal connecting the air reactor to the fuel reactor. Also, some N_2 flow comes from this loop seal. In addition, gas coming from the carbon stripper is added in the upper part of the dense bed. The carbon stripper was designed to separate unconverted char particles from the oxygen carrier. However, the carbon stripper was not effective for pelletized biomass and it could not be included in an industrial BCLG unit. To approach the conditions existing in a unit without carbon stripper, this reactor was fluidized with inert N_2 to prevent the char gasification in this device. Gaseous flows in these inlet points to the fuel reactor are detailed in Table S2.

Main properties of ilmenite particles are summarized in Table S3. Also, this table shows the kinetic parameters for ilmenite reactions with CO, H_2 and CH_4 which was determined by Abad et al.[S3] in a thermogravimetric analyzed (TGA). The main properties of the biomass after devolatilization (described as devolatilized pellets) and its gasification kinetic parameters are shown in Table S4, which were also determined by TGA tests.[S4] This table also shows the volatiles composition, which was determined by a modification of the model presented by Matthesius et al.,[S5] to predict the main products presented by Neves et al.[S6] for biomass pyrolysis at the high-temperature interval used in BCLG process. Due to the kinetics for the ilmenite reduction with C2, C3 or higher hydrocarbons in the volatile matter was not known, the model was modified to consider CH_4 as the only hydrocarbon; see Table S4.

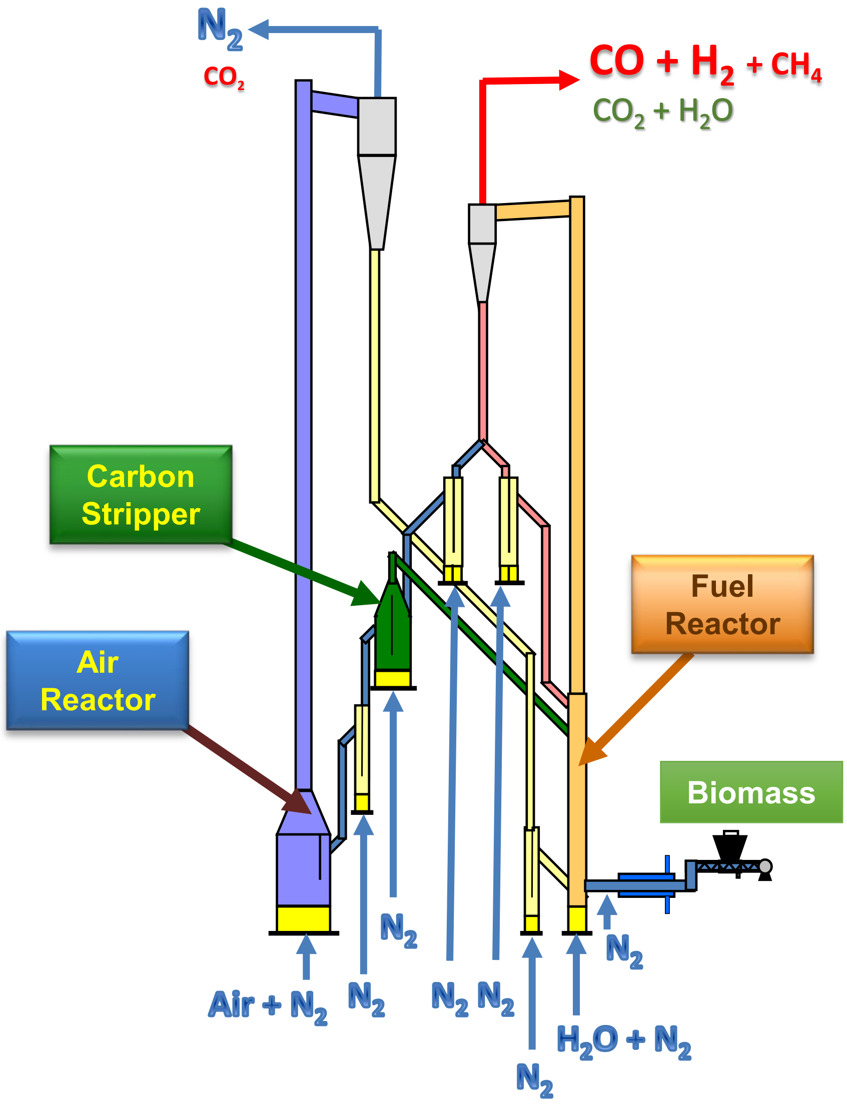

Figure S1. Scheme of the BCLG unit where tests in Table S3 were performed.

Table S1. Main dimensions of the fuel reactor.

**Reactor geometry**	Symbol	Value
Height bottom part (m)	H_{bottom}	1.2
Height upper part (m)	H_{up}	2.8
Diameter bottom part (m)	d_{bottom}	0.102
Diameter upper part (m)	d_{up}	0.081
Height of the biomass feeding (m)	H_{fuel}	0.05
Height of solids from loop seal (m)	H_{LS}	0.10
Height of the gas from CS (m)	H_{CS}	1.0
Number of nozzles (gas distributor)	N_{nz}	72

Table S2. Operating conditions for the selected tests to validate the fuel reactor model.[S1,S2]

								Gas flows (m^3/h, STP)			
**Biomass-Test**	**Power**	**Fuel feeding rate**	**Temperature in FR**	**Pressure drop**	**Solids circulation rate**	**Oxygen ratio in FR**	**Carbon conversion in FR**	**H_2O to FR**	**N_2 from fuel feeding**	**N_2 from LS**	**total N_2 from CS**
	P_{th} (kW)	F_{fuel} (kg/h)	T_{FR} (ºC)	ΔP_0 (kPa)	F_s (kg/h)	λ_{FR}	$X_{C,FR}$ (%)	$F_{g,in}$	$F_{g,fuel}$	$F_{g,LS}$	$F_{g,CS}$
WSP-1	18.3	3.84	882	7.9	70	0.30	79.8	2.99	0.50	0.03	4.80
WSP-2	18.3	3.83	897	7.3	43	0.29	91.3	2.49	1.50	0.30	4.25
WSP-3	18.3	3.83	910	9.2	79	0.32	90.3	2.49	1.50	0.30	4.25
WSP-4	19.0	3.98	891	6.5	114	0.23	81.9	2.49	1.50	0.60	6.80
WSP-5	19.0	3.98	986	6.1	445	0.20	82.0	2.49	1.50	0.60	6.80
WSP-6	19.0	3.98	983	6.2	435	0.14	80.5	2.49	1.50	0.60	6.80
WSP-7	19.0	3.98	978	7.7	438	0.14	72.1	2.49	1.50	0.45	6.69
WSP-8	19.0	3.98	879	4.7	100	0.06	73.7	2.49	1.50	0.60	6.60
WSP-9	18.5	3.88	947	5.9	98	0.39	93.3	2.49	1.50	0.70	6.95
WSP-10	18.5	3.88	939	7.3	150	0.44	89.2	2.49	1.50	0.56	6.95
WSP-11	18.5	3.88	934	7.0	101	0.42	91.8	2.49	1.50	0.75	6.97
WSP-12	18.5	3.88	948	10.4	110	0.37	97.6	3.11	1.50	0.90	4.15
WSP-13	18.5	3.88	936	8.0	160	0.37	86.0	4.11	1.50	0.92	4.17
WSP-14	18.5	3.88	928	7.5	160	0.32	97.8	2.49	1.50	0.55	7.43
PFR-1	21.6	4.33	916	9.9	205	0.06	80.5	2.49	1.50	0.93	4.15
PFR-2	21.6	4.33	920	15.7	205	0.44	84.3	2.49	1.50	0.93	7.15
PFR-3	21.6	4.33	935	6.9	124	0.24	78.4	2.49	1.50	0.90	7.31

Table S3. Main properties of ilmenite particles.

**Physical properties of ilmenite**	Symbol	Value		
Mean particle diameter (mm)	d_{OC}	0.225		
Sphericity (-)	φ_{OC}	1		
Apparent density (kg/m^3)	ρ_{OC}	3050		
Oxygen transport capacity (%)	R_{OC}	3.7		
**Kinetic parameters**[S3]				
Molar density (mol/m^3)	ρ_m	13590		
Grain radius (m)	r_g	$1.25 \cdot 10^{-6}$		
		H_2	CO	CH_4
Stoichiometric coeff. (-)	b	1.45	1.45	5.78
Preexponential factor (mol^{1-n} m^{3n-2} s^{-1})	$k_{0,r}$	$6.2 \cdot 10^{-2}$	$1.0 \cdot 10^{-1}$	9.8
Activation energy (kJ/mol)	$E_{a,r}$	65.0	80.7	135.9
Reaction order (-)	n	1	0.8	1

Table S4. Main properties of pelletized biomasses WSP and PFR.

**Physical properties of devolatilized pellets**	Symbol	WSP	PFR
Pellet diameter (mm)	d_f	4.8	4.8
Sphericity (-)	φ_f	0.78	0.78
Apparent density (kg/m^3)	ρ_f	493	1040
**Proximate analysis (wt%)**			
Moisture		10.3	3.3
Ash		6.2	1.3
Volatile matter		69.8	77.2
Fixed carbon		13.7	18.3
**Ultimate analysis (wt%)**			
C		41.4	51.5
H		5.2	5.8
N		0.4	0.3
S		0.1	0.0
O		36.4	37.8
**Volatile composition (g per 100 g of fuel)**[S5,S6]			
CH_4		8.8281	10.8152
CO		28.0479	48.3155
CO_2		33.2140	16.0671
H_2		3.3739	3.2187
H_2O		6.0441	1.6192
NH_3		0.4857	0.3646
H_2S		0.1063	0.0000

**Gasification kinetic parameters with H_2O**[S4]			
Reaction order (-)	n	1	0
Preexponential reaction factor (s^{-1} atm^{-1})	$k_{0,1}$	$4.93\cdot10^{2}$	$6.90\cdot10^{1}$
Activation energy of reaction (kJ/mol)	$E_{a,1}$	107.1	89.9
Preexponential adsorption factor H_2O (atm^{-1})	$k_{0,2}$	$9.41\cdot10^{-3}$	$3.10\cdot10^{-2}$
Activation energy adsorption H_2O (kJ/mol)	$E_{a,2}$	-53.1	-41.4
Preexponential adsorption factor H_2 (atm^{-1})	$k_{0,3}$	$2.27\cdot10^{-5}$	$2.10\cdot10^{-6}$
Activation energy adsorption H_2 (kJ/mol)	$E_{a,3}$	-126.7	-143.7
**Gasification kinetic parameters with CO_2**[S4]			
Reaction order (-)	n	1	0
Preexponential reaction factor (s^{-1} atm^{-1})	$k_{0,1}$	$4.76\cdot10^{1}$	$1.40\cdot10^{2}$
Activation energy of reaction (kJ/mol)	$E_{a,1}$	84.9	101.4
Preexponential adsorption factor CO_2 (atm^{-1})	$k_{0,2}$	$1.38\cdot10^{-4}$	$2.70\cdot10^{-3}$
Activation energy adsorption CO_2 (kJ/mol)	$E_{a,2}$	-94.2	-64.1
Preexponential adsorption factor CO (atm^{-1})	$k_{0,3}$	$7.13\cdot10^{-7}$	$3.10\cdot10^{-7}$
Activation energy adsorption CO (kJ/mol)	$E_{a,3}$	-155.2	-161.5

The hypotheses considered for the model were: (1) steady state; (2) isothermal bed at macroscopic level; (3) perfect mixing of the solids in the dense bed; (4) plug flow of gas in all zones of the reactor but lateral exchange of gas between bubbles and emulsion in the dense bed; (5) gas stagnant in the annulus of the dilute region, that is, the gas flows only through the core; (6) plug flow up of solids through the core in the dilute region with lateral flow of solids from the core to the annulus; and (7) no existence of particle fragmentation or attrition. The modelling of the fuel reactor was divided in two fields: fluid dynamics and mass balances. At this point, it is remarkable that the gas velocity, u_g, was not constant with the reactor height because the gas entering from the carbon stripper, the gases from the pyrolysis and gasification of fuel, the gas expansion during the conversion of hydrocarbons in the volatile matter and the gas expansion due to the pressure drop in the reactor. This affected to the fluid dynamics of the fluidized bed and depended on the reaction rate of gases with the oxygen carrier and char. Therefore, fluid dynamics and mass balances in the reactor had to be solved simultaneously.

1.2. Fluid dynamics. The mixing of gas and solid particles influences the performance of a fluidised–bed reactor. Therefore, the model must consider the gas and solids flows inside the

reactor and the gas–solids mixing patterns in the different regions in which it can be divided. The model is one–dimensional, with the main dimension being the axial direction, but it takes into account lateral exchange of solids in the dilute region between the core and an annulus close to the reactor wall. Thus, the fluid dynamics is considered to be a 1.5-Dimensional macroscopic model based on empirical and semi-empirical expressions[S7], but modified to consider the specific conditions for biomass gasification in the 20 kW_{th} BCLG unit.[S1,S2] Thus, the fuel reactor is a fluidised bed working at the slugging regime in the bottom bed and the high–velocity regime in the upper part, after gas coming from the carbon stripper is joined to the gas from the bottom bed. In addition, pelletized biomass was used as fuel. The reactor was divided into two vertical zones with respect to axial concentration and backmixing of solids; see Table S1:

1) A dense bed with a high and roughly constant solids concentration. In this case, this zone corresponded to the bottom part, 1.2 m length, with a higher diameter than the riser. This zone has a high L/D ratio and relatively low velocity, which makes it operate in the slugging regime. A bubble-emulsion structure was considered, with bubbles being able to occupy the entire cross section of the fuel reactor, and gas exchange between these phases.

2) A dilute region above the dense bed, where there is a pronounced decay in solids concentration with height. The dilute region is composed by the transport phase, forming a core/wall–layer flow structure, with the solids backmixing mainly at the reactor walls, F_w, and a net flow up of solids in the core, F_c.

1.2.1. Fluid dynamics in the dense bed. The dense bed is defined as the zone located at the bottom of the bed characterized by a roughly constant solids concentration when averaged in time. The total gas flow is divided in three parts: (1) the flow in the particulate or emulsion phase at the minimum fluidization velocity, u_{mf}; (2) the visible bubble flow, u_{vis}, related to the gas in the bubbles at the rising velocity of the bubbles; and (3) the gas throughflow, u_{tf}, corresponding

to an excess of inlet gas over the gas in the emulsion and bubbles, which pass through the bubbles. Thus, the total gas flow, u_g, is divided following the equation:

$$u_g = (1-\delta_b)u_{mf} + u_{vis} + u_{tf} \tag{S1}$$

A gas exchange between bubbles (u_{vis} and u_{tf}) and emulsion (u_{mf}) is considered allowing the exchange of products and reactants between these phases. The model assumes that the emulsion phase remains under minimum fluidization condition. Thus, the flow in excess over the minimum gas velocity, u_{mf}, is divided between the gas in the bubbles, u_{vis}, and the throughflow, u_{tf}. The minimum fluidization velocity, u_{mf}, was calculated by:[S8]

$$\mathrm{Re}_{p,mf} = \frac{u_{mf}\rho_g d_{OC}}{\mu_g} = \sqrt{27.2^2 + 0.0408\mathrm{Ar}_{OC}} - 27.2 \tag{S2}$$

The porosity at the minimum fluidization conditions, ε_{mf}, was calculated by:[S9]

$$\varepsilon_{\mathrm{mf}} = 0.586\varphi^{-0.72}\mathrm{Ar}_{OC}^{-0.029}\left(\frac{\rho_g}{\rho_{OC}}\right)^{0.021} \tag{S3}$$

The visible bubble flow and the throughflow were written:

$$u_{vis} = \psi\left(u_g - u_{mf}(1-\delta_b)\right) \tag{S4}$$

$$u_{tf} = (1-\psi)\left(u_g - u_{mf}(1-\delta_b)\right) \tag{S5}$$

Ψ being the ratio of the visible bubble flow, u_{vis}, to the total flow through the bubbles, $u_{vis} + u_{tf}$, and it was calculated as:

$$\psi = f_b\left(z + 4\sqrt{A_0}\right)^{0.4} \tag{S6}$$

The parameter A_0 is the gas-distributor area per nozzle. The function f_b was calculated from the following equation:

$$f_b = \frac{0.26+0.70e^{-3300d_{OC}}}{\left(0.15+u_g-u_{mf}\right)^{1/3}} \tag{S7}$$

The bubble fraction in the dense bed was:

$$\delta_b = \frac{u_{vis}}{u_{vis}+u_{b\infty}} \tag{S8}$$

With the assumption of the minimum fluidization porosity in the emulsion phase, the single bubble velocity, $u_{b\infty}$, was:

$$u_{b\infty} = 0.71\sqrt{gd_b} \tag{S9}$$

the bubble size being calculated with the correlation by:[S10]

$$d_b = 0.54\left(u_g - u_{mf}\right)^{0.4}\left(z + 4\sqrt{A_0}\right)^{0.8} g^{-0.2} \tag{S10}$$

Once the fraction of bubbles in the bed, δ_b, was obtained, the average bed porosity at a fixed position in the dense bed can be obtained as:

$$\varepsilon_b = (1 - \delta_b)\varepsilon_{mf}+\delta_b \tag{S11}$$

However, under the design and operating conditions of the CLG unit, the model predicts a fast growing of the bubble size until its diameter reaches the reactor diameter. Under these conditions, it is predicted a slugging fluidization regime, which is characterized by the presence of big bubbles and a relatively low average concentration of solids. In addition, solids are continuously ascending and descending in block along the length of the bottom bed. To contemplate this regime, the original model[S7,S11] was modified considering that the amount of solids was homogeneously distributed along the dense bed, which is true when this property was averaged in time. Thus, the emulsion fraction was calculated from the pressure drop in the bottom bed, ΔP_b:

$$f_{\mathrm{s,b}} = 1 - \delta_b = \frac{\Delta P_b}{\rho_{oc}(1-\varepsilon_{\mathrm{mf}})H_b g} = \frac{C_b}{\rho_{oc}(1-\varepsilon_{\mathrm{mf}})} \tag{S12}$$

The pressure drop in the bottom bed was determined during the calculation procedure considering that the sum of pressure drop in the bottom bed, ΔP_b, and in the dilute region, ΔP_d, is the total pressure drop in the reactor, ΔP_0:

$$\Delta P_0 = \Delta P_b + \Delta P_d \tag{S13}$$

2.2.2. Fluid dynamics in the dilute region. The dilute region starts at the upper limit of the dense bed, and it is characterised by a decrease in the solids concentration with the reactor height. In the 20 kW_{th} BCLG unit, a low pressure drop in this region is observed, which corresponds to the low solids concentration existing in the transport phase. Thus, the transport phase is characterised by a disperse zone with the solids being distributed in a core-annulus structure. There is a net flux of solids upwards in the core, and downwards in the annulus. Thus, the backmixing in the transport phase occurs mainly at the reactor walls. The gas flow up through the core, whereas it is assumed to be stagnant in the annulus, near the walls of the reactor. Because of the small length of this zone compared to industrial reactors, a saturation value of the annulus thickness was considered for the whole diluted region:

$$\delta_w = \delta_{\mathrm{w,sat}} = 0.06456 d_{up} \tag{S14}$$

The flow rate of solids from the dense bed to the transport phase, $F_{0,\mathrm{i}}$, was obtained by:[S12]

$$F_{0,i} = 131.1\left[\frac{A_c u_g}{\varepsilon_b}\rho_g\left(\frac{\mathrm{Re_{p,i}}}{\mathrm{Ar_i}}\right)^{0.31}\right]_{H_b} \tag{S15}$$

The solids concentration in the transport phase was taken from the flow rate of entrained solids from the dense region, $F_{0,\mathrm{i}}$:

$$C_{tr,i,H_b} = \frac{F_{0,i}}{A_{c,H_b}\left(u_{g,H_b} - u_{t,i}\right)} \tag{S16}$$

The decay in solids concentration in the transport phase was given by the decay factor *K*:

$$\frac{\mathrm{d}C_{tr,i}}{\mathrm{d}z} = -K_i C_{tr,i} \tag{S17}$$

$$K_i = min\left\{-0.1533,\ \frac{0.23}{u_g - u_{t,i}}\right\} \tag{S18}$$

The terminal velocity was calculated using the following correlations for oxygen carrier:[S13]

$$u_{t,OC} = u^*_{t,OC}\left(\frac{\rho_g^2}{\mu_g(\rho_{OC}-\rho_g)g}\right)^{-1/3} \quad \text{being} \quad u^*_{t,OC} = \left(\frac{18}{\mathrm{Ar}_{OC}^{2/3}} + \frac{2.335 - 1.744\varphi_{OC}}{\mathrm{Ar}_{OC}^{1/6}}\right)^{-1} \tag{S19}$$

Char pellets were considered coarse particles in multi-solid fluidized bed, i.e. highly diluted in oxygen carrier particles.[S14] Thus, the current terminal velocity of char fuel pellets, $u_{t,f}$, was lower than the terminal velocity for isolated pellets, which was estimated to be about 10 m/s.

$$\frac{18}{d_f}\left[\frac{1+Re_f^{0.687}}{Re_f}\right]\frac{\rho_g}{\rho_f}u_{t,f}^2 + \frac{3G_s(1+d_{OC}/d_f)^2}{2\ \rho_f d_f}\left(u_{t,f} - u_{t,OC}\right) = g \tag{S20}$$

The decay factor *K* is not constant along the dilute phase, because the gas velocity, u_g, changes with the height in the reactor. The concentration of ilmenite and char in the transport phase was calculated separately by Eq. (S17), being $C_{tr,i}$ the concentration of the solids *i* considered, i.e. ilmenite or char, and the total concentration in the transport phase the sum of the concentration of char and ilmenite:

$$C_{tr,H_b} = C_{tr,OC,H_b} + C_{tr,C,H_b} \tag{S21}$$

Particles entrained up through the dispersed region can be separated to the annulus near the reactor wall or externally recirculated through the cyclone after having reached the exit duct height. From the solids concentration in the core, the upwards solids flow, F_c, can be obtained as:

$$F_c = C_{tr}A_c\left(u_g - u_t\right) \tag{S22}$$

The transport phase contributes to the externally recirculated solid flow, F_s, which can be obtained from the entrainment probability, p_{ent}, or the backflow ratio, k_b, and the solid flow at the exit zone, which was assumed to be at H_r:

$$p_{ent} = \frac{1}{k_b+1} = \frac{F_s}{F_{c,H_r}} \tag{S23}$$

Despite its importance, little information is available on the magnitude of the entrainment probability, p_{ent}, or the backflow ratio, k_b.[S15,S16] Factors as the geometry of the exit zone, the inlet area of the cyclone, the slip velocity or the value of the solids net flow can affect this parameter. Recently, some data are available for entrainment for systems applied to chemical looping units.[S17] However, uncertainty on the p_{ent} value is still high, and it will be determined from model results in this work.

Finally, the mixing gas behaviour in the dilute region was considered. This phenomenon was taken into account by the use of a contact efficiency parameter, ξ_{g-s}, between gas and solids in the dilute region, calculated by:[S18]

$$\xi_{g-s} = 1 - 0.75\left(\frac{C_{dil}}{C_{b,H_b}}\right)^{0.4} \tag{S24}$$

C_{dil} being the solids concentration in the dilute region and $C_{b,Hb}$ the solids concentration in the upper limit of the dense bed. The contact efficiency parameter was applied on the reaction rate calculated at each axial position in the dilute region.

2.3. Mass balances. Mass balances for the different reacting compounds and products were developed for the phases in the dense bed and the dilute region. The pathway for biomass conversion with the oxygen carrier was considered to happen in two steps. First, devolatilization and gasification to produce gaseous compounds; see reactions (SR1-SR3). The composition of volatile matter for WSP and PFR are shown in Table 4. Also, due to the devolatilization times

for pellets are higher than the typical mixing time of solids in the dense bed, it is assumed that devolatilization happens homogeneously through the whole dense bed. Second, H_2, CO and CH_4 react with the oxygen carrier; see reactions (SR4) and (SR5). The pathway for reaction of methane with the oxygen carrier considers that H_2O is a primary product during the reaction with the metal oxide.[S19,S20] Thus, the CH_4 conversion was considered to happen in two steps: first towards CO and H_2O and later CO reacts towards CO_2. Moreover, the model considers the non-catalytic water–gas shift reaction kinetics.[S19,S21]

$$Biomass \xrightarrow{\Delta} Volatiles(CO, H_2, CH_4, \ldots) + char(C) \quad \text{(SR1)}$$

$$char(C) + H_2O \longrightarrow CO + H_2 + ash \quad \text{(SR2)}$$

$$char(C) + CO_2 \longrightarrow 2\,CO + ash \quad \text{(SR3)}$$

$$CO \,/\, H_2, \ldots + nMe_xO_y \longrightarrow CO_2 \,/\, H_2O + nMe_xO_{y-1} \quad \text{(SR4)}$$

$$C_nH_m, \ldots + (n + \tfrac{m}{2})Me_xO_y \longrightarrow n\,CO + \tfrac{m}{2}H_2O + (n + \tfrac{m}{2})Me_xO_{y-1} \quad \text{(SR5)}$$

2.3.1. Mass balances in the dense bed. In the dense bed, a gas exchange between bubbles (u_{vis} and u_{tf}) and emulsion (u_{mf}) is considered allowing the exchange of products and reactants between these phases by diffusive and/or bulk flow mechanism. Indeed, as the gas suffers a volumetric expansion during the char gasification and conversion of hydrocarbons, some of the gas in the emulsion must move to the bubble phase to maintain the minimum fluidization condition in the emulsion phase. Considering all the above assumptions, the mass balances were given by the following differential equations for each gas *i* (CO, H_2, CO_2, H_2O and CH_4) in the emulsion and bubble phases, respectively:

$$\frac{dF_{e,i}}{dV} = \frac{d[(1-\delta_b)u_{mf}C_{e,i}]}{dz} = -(1-\delta_b)\left[\Sigma(-\bar{r}_{g,i})_{OC} + \Sigma(-\bar{r}_{g,i})_{char}\right]_e - \delta_b k_{be}(C_{e,i} - C_{b,i}) + \frac{F_{vol,i}}{dV} - y_{e,i}\frac{dF_{exc}}{dV} - \frac{dF_{WGS,i}}{dV} \quad \text{(S25)}$$

$$\frac{dF_{b,i}}{dV}=\frac{d[(u_{vis}+u_{tf})C_{b,i}]}{dz}=\delta_b k_{be}\left(C_{e,i}-C_{b,i}\right)+y_{e,i}\frac{dF_{exc}}{dV}-\frac{dF_{WGS,i}}{dV} \quad \text{(S26)}$$

The first term in the right side on Eq. (S25) represents the amount of gas *i* consumed or generated in the differential volume element d*V*. $\sum\left(-\bar{r}_{g,i}\right)_{OC}$ is the net consumption or generation rate of the gas *i* by reaction with the oxygen carrier in the emulsion phase. $\sum\left(-\bar{r}_{g,i}\right)_{char}$ is the net consumption or generation of the gas *i* by the char gasification. The second term is the net gas amount of gas *i* flowing from the emulsion to the bubble by gas diffusion. The third term is the gas generation from devolatilization. It is assumed a homogeneous devolatilization in the dense bed, and that it happens in the emulsion, i.e. where the solids particles are. Then, the fourth term refers to the excess of gas in the emulsion that moves to the bubbles because of the gas expansion produced in the differential volume. Finally, the fifth term is the amount of gas *i* reacted by WGS. Similar descriptions can be done for the mass balance in the bubble phase -see Eq. (S26)- excepting that in this phase there is not reactions involving solids. These equations allow to determine the concentration of gas *i* in both phases: emulsion and bubbles. The gas exchange between bubbles and emulsion was determined by the bubble–emulsion gas exchange coefficient, k_{be}, which was obtained by:[S22]

$$k_{be}=1.631\;u_g\mathrm{Sc}^{0.37} \quad \text{(S27)}$$

2.3.2. Mass balances in the dilute region. Above the dense bed starts the dilute region, which is characterized by a strong decay on solids concentration, as it is described in Eq. (S17). In the dilute region, the mass balance for each gas in a differential element of bed yields:

$$\frac{dF_{dil,i}}{dV}=\frac{d[u_g C_{dil,i}]}{dz}=-\xi_{\text{g-s}}\left[\sum\left(-\bar{r}_{g,i}\right)_{OC}+\sum\left(-\bar{r}_{g,i}\right)_{char}\right]_{tr}-\frac{dF_{WGS,i}}{dV} \quad \text{(S28)}$$

In this case, the first term in the right side on Eq. (S28) represents the amount of gas *i* consumed or generated in the differential volume element d*V* by reaction of gases with the oxygen carrier

and char particles in the transport phase. ξ_{g-s} is the contact efficiency between gas and solids in the freeboard given by Eq. (S24). The second term is the amount of gas *i* reacted by WGS.

Solids in the transport phase have a net flow upwards through the core, considered in plug flow. Thus, the solids conversion increases as the solids flow up through the core in the transport phase. Solids conversion, as a function of axial location in the transport phase was calculated using the following equation for both oxygen carrier and char particles:

$$\frac{dX_s}{dz} = \frac{dX_s dt}{dt\ dz} = \frac{dX_s}{dt}\frac{1}{u_{s,c}} \tag{S29}$$

$u_{s,c}$ being the velocity of solids in the core

$$u_{s,c} = u_g - u_t \tag{S30}$$

2.3.3. Oxygen carrier reactivity. The changing grain size model with uniform reaction in the particle was used to determine the reaction rate of the oxygen carrier particles. Every grain reacts following the shrinking core model (SCM) controlled by chemical reaction. The equations that describe this model are the following:[S3]

$$\frac{t}{\tau_i} = 1-(1-X_{OC,i})^{1/3} \qquad \tau_i = \frac{\rho_m r_g}{b_i k_{r,i} C_{p,i}^n} \tag{S31}$$

Ilmenite showed an increase in its reactivity through redox cycles during the so-called activation process.[S23] A fast activation was observed in continuous operation in a 20 kW_{th} unit, whereas the oxygen transport capacity was maintained roughly constant.[S1,S2] In this work, activated ilmenite was considered as the oxygen carrier material for coal combustion. The physical properties and kinetic parameters for reduction of ilmenite with CH_4, CO and H_2 of the activated ilmenite particles are shown in Table S3. The dependence on the temperature of the kinetic constant was assumed to be Arrhenius type, as follows:

$$k_r = k_{0,r} e^{-E_{a,r}/R_g T} \tag{S32}$$

The gas concentration was assumed to be constant throughout the particle and equal to the gas concentration at the external particle surface, C_p=C_{ps}. The gas concentration in the external particle surface, C_{ps}, can be obtained by a mass balance to the whole particle:

$$\left(-\bar{r}_{g,i}\right)_{OC} = \frac{\rho_s R_{OC}}{b_i M_O}\left(\frac{4}{3}\pi r_p^3\right)\left[\frac{\mathrm{d}\left(X_{OC}(t) - \bar{X}_{OC,in}\right)}{\mathrm{d}t}\right]_i = k_{g,i}\left(4\pi r_p^2\right)\left(C_{z,i} - C_{ps,i}\right) \tag{S33}$$

The second term in Eq. (S33) is the gas *i* reacted in the whole particle and the third term is the gas flow from the bulk gas to the particle by external diffusion. Thus, it was considered the gas concentration at the external particle surface, C_{ps}, was lower than the gas concentration in the bulk gas, C_z, because the external diffusion through the gas film around the particle. The mass transfer coefficient for each gas, $k_{g,i}$, was obtained by:[S24,S25]

Dense bed: $$\mathrm{Sh} = \frac{k_{g,i} d_{OC}}{D_{g,i}} = 2\varepsilon_{\mathrm{mf}} + 0.117\mathrm{Ar}^{0.39}\mathrm{Sc}^{1/3} \tag{S34}$$

Dilute region: $$\mathrm{Sh} = \frac{k_{g,i} d_{OC}}{D_{g,i}} = 2\varepsilon_z + 0.69\mathrm{Re}_p^{1/2}\mathrm{Sc}^{1/3} \tag{S35}$$

The reactivity of the particles was obtained deriving Eq. (S31):

$$\left[\frac{\mathrm{d}(X_{OC}(t))}{\mathrm{d}t}\right]_i = \frac{3}{\tau_i}[1 - X_{OC}]^{2/3} \tag{S36}$$

The average reaction rate of the oxygen carrier at each height in the reactor to be used in Eqs. (S25) and (S28), $\left(-\bar{r}_{g,i}\right)_{OC}$, was calculated considering the stoichiometric coefficient of the gas-solid reaction, b_i:

$$\left(-\bar{r}_{g,i}\right)_{OC} = \frac{\left(-\bar{r}_{OC,i}\right)}{b_i} \tag{S37}$$

The fuel reactor model considers that the oxygen carrier could not be fully oxidized in the air

reactor, and particles can be introduced into the fuel reactor with a mean conversion of the carrier for the reduction reaction, $\overline{X}_{OC,in}$, higher than 0. In a previous work, it was determined that ilmenite particles react with the rate corresponding to the current conversion.[S26] Thus, the maximum variation possible in conversion of the oxygen carrier is $\left(1-\overline{X}_{OC,in}\right)$. Consequently, the reaction rate of a particle entering to the fuel reactor is calculated for its conversion, i.e. $\overline{X}_{OC,in}$. The reaction rate of particle changes with the reacting time until it comes out of the reactor, or it was completely reduced, i.e. $\overline{X}_{OC}$=1.

To obtain the average reaction rate of the oxygen carrier, $\left(-\overline{r}_{OC,i}\right)$, it was assumed a perfect mixing of the solids in the dense bed. Thus, the residence time distribution curve of the solids, E(t), is given by the following equation:

$$E(t)=\frac{1}{t_{mr}}e^{-t/t_{mr}} \qquad \text{(S38)}$$

where t_{mr} is the mean residence time of particles in the whole reacting zone of the fluidized–bed reactor. The conversion as a function of the residence time of a particle in the reactor is obtained taking into account the mean conversion of the carrier at the reactor inlet and the conversion variation after this time

$$X_{OC}(t)=\overline{X}_{OC,in}+\left[1-\left(1-\frac{t+t_r}{\tau_m}\right)^3\right] \qquad \text{(S39)}$$

and the mean conversion of the particles, $\overline{X}_{OC,out}$, can be obtained as

$$\left(1-\overline{X}_{OC,out}\right)=\int_0^{\tau_m}(1-X_{OC}(t))E(t)dt \qquad \text{(S40)}$$

The value of t_r was defined as the reacting time of an oxygen carrier particle from zero conversion until the inlet average conversion, i.e. $\overline{X}_{OC,in}$. Thus, the t_r value can be obtained as:

$$t_r\text{=}\tau\left[1\text{-}(1\text{-}\overline{X}_{OC})^{1/3}\right] \qquad \text{(S41)}$$

The mean reacting time, τ_m, is determined by an iterative process to obtain from Eqs. (S39) and (S40) the corresponding mean conversion at the reactor outlet, $\overline{X}_{OC,out}$, that fit the mean conversion obtained from the mass balance to the whole reactor.

$$F_{OC}R_{OC}\Delta X_{OC} = \lambda_{FR}M_O\Omega_{fuel}F_{fuel} \tag{S42}$$

ΔX_{OC} being the variation of the mean conversion of solids in the reactor ($\Delta X_{OC} = \overline{X}_{OC,out} - \overline{X}_{OC,in}$), λ_{FR} the oxygen-to-fuel ratio transferred in the fuel reactor and Ω_{fuel} the oxygen demand of the solid fuel. λ_{FR} is defined as the ratio of the flow of oxygen taken up by the fuel to the amount of oxygen that would have been taken up if conversion of fuel was complete to CO_2 and H_2O.

Once the distribution of the conversions was obtained –Eq. (S40)–, the average reaction rates of the oxygen carrier in the reactor can be obtained as:

$$\left(-\bar{r}_{OC,i}\right) = \frac{\rho_{OC}R_{OC}}{M_O}(1-\varepsilon_z)\int_{t_r}^{\tau}\left[\frac{\mathrm{d}(X_{OC}(t))}{\mathrm{d}t}\right]_i E(t)dt \tag{S43}$$

Eq. (S43) has been expressed to consider that the oxygen carrier is introduced into the reactor with a mean conversion for the reduction reaction, $\overline{X}_{OC,in}$, higher than 0.

2.3.4. Fuel conversion. When the fuel is fed to the fuel reactor different physical and chemical processes happen. Devolatilization comes first, where the volatile matter is evolved to the gaseous stream. Eq. (S25) assumes the homogeneous devolatilization of biomass pellets in the dense bed due to its relatively high size and devolatilization time. The remaining carbon in char is gasified with H_2O and CO_2; reactions (SR2) and (SR3). Kinetic parameters for gasification of the biomasses used in the 20 kW_{th} BCLG unit, namely WSP and PFR, are shown in Table S4. Different behaviour has been found for the gasification of char from WSP and PFR in the pellet form.[S4] The homogeneous model has been found for WSP to describe the gasification process.

Thus, the reaction rate for the gasification of WSP with H_2O and CO_2 was calculated considering that the reaction order for the conversion of solid is n=1:

$$-\frac{1}{m_C}\left[\frac{\mathrm{d}m_C}{\mathrm{d}t}\right]_{H_2O} = \frac{1}{1-X_C}\left[\frac{\mathrm{d}X_C}{\mathrm{d}t}\right]_{H_2O} = \frac{k_{1,H_2O}P_{H_2O}}{1+k_{2,H_2O}P_{H_2O}+k_{3,H_2O}P_{H_2}} \quad \text{(S44)}$$

$$-\frac{1}{m_C}\left[\frac{dm_C}{dt}\right]_{CO_2} = \frac{1}{1-X_C}\left[\frac{dX_C}{dt}\right]_{CO_2} = \frac{k_{1,CO_2}P_{CO_2}}{1+k_{2,CO_2}P_{CO_2}+k_{3,CO_2}P_{CO}} \quad \text{(S45)}$$

The net consumption or generation of the gas i by the char gasification, $\left(-\bar{r}_{g,i}\right)_{char}$ to be used in Eqs. (S25) and (S28), was given by

$$\left(-\bar{r}_{g,i}\right)_{char} = \frac{C_C\rho_s(1-\varepsilon_z)}{M_C}\left[\frac{1}{1-X_C}\left[\frac{\mathrm{d}X_C}{\mathrm{d}t}\right]\right]_i \quad \text{(S46)}$$

In the case of PFR, the gasification rate was constant with conversion of solids following a reaction order n=0:

$$-\frac{1}{m_{C,0}}\left[\frac{dm_C}{dt}\right]_{H_2O} = \left[\frac{dX_C}{dt}\right]_{H_2O} = \frac{k_{1,H_2O}P_{H_2O}}{1+k_{2,H_2O}P_{H_2O}+k_{3,H_2O}P_{H_2}} \quad \text{(S47)}$$

$$-\frac{1}{m_{C,0}}\left[\frac{dm_C}{dt}\right]_{CO_2} = \left[\frac{dX_C}{dt}\right]_{CO_2} = \frac{k_{1,CO_2}P_{CO_2}}{1+k_{2,CO_2}P_{CO_2}+k_{3,CO_2}P_{CO}} \quad \text{(S48)}$$

$m_{C,0}$ being the mass of carbon initially present in the partially converted char in the fuel reactor, which is a function of the char conversion:

$$m_{C,0} = \frac{m_C}{X_C} \quad \text{(S49)}$$

The generation rate of gasification products must be calculated considering the char distribution time in the reactor, similarly to what was used for the oxygen carrier in Eq. (S43):

$$\left(-\bar{r}_{g,i}\right)_{char} = \frac{C_C\rho_s(1-\varepsilon_z)}{M_C}\int_0^{\tau}\left[\frac{d(X_C(t))}{\mathrm{d}t}\right]_i E(t)dt \quad \text{(S50)}$$

A relevant parameter to calculate the generation of gasification products is the carbon concentration in the bed, C_C, which is a function of the region in the bed. C_C is constant in the dense bed, but varies with the reactor height according to the char conversion variation -Eq. (S29)- and the decay of oxygen carrier and char concentration -Eq. (S17)- in the dilute region.

2.4. Boundary and initial conditions. The boundary conditions to solve the above mass balances are the following:

a) At the bottom of the bed, the gas flow of each compound *i* is the flow at the reactor inlet.

$$F_i|_{z=0} = F_{i,in} \tag{S51}$$

b) The gas flow from the loop seal in incoming solids, the fuel feeding system and the stream from the carbon stripper is added at their corresponding positions; see Tables S1 and S2.

c) The dense bed has a height of 1.2 m, and the riser is 2.8 m tall.

Moreover, the following initial conditions should be fulfilled:

a) The amount of solids in the fuel reactor should be that required to give the pressure drop initially assumed. Thus, the height of the dense bed is calculated to fulfil the amount of solids required.

$$\Delta P = \int_0^{H_b} C_b \rho_s \mathrm{g} dz + \int_{H_b}^{H_r} C_{dil} \rho_s \mathrm{g} dz \tag{S52}$$

b) The oxygen supplied by the oxygen carrier should be equal to the oxygen reacted with the reacting gases from pyrolysis and gasification. This criterion is used for the convergence of the variation of the conversion of the oxygen carrier in the reactor, ΔX_{OC}. Thus, for low ΔX_{OC} values the average reactivity is high and oxygen taken by the fuel is higher than the oxygen supplied by the oxygen carrier. The reactivity decreases as ΔX_{OC} increases. Thus, a solution

can be found for the oxygen balance in the fuel reactor. More information about the variation of the average reactivity with the variation of the solids conversion can be found elsewhere.[S27]

c) The flow of solids exiting the fuel reactor, both oxygen carrier ($F_{FRout,OC}$) and unconverted char ($F_{FRout,C}$), is a function of the solids flow in the core at the exit position H_r, i.e. $F_{c,Hr,OC}$ and $F_{c,Hr,C}$, as well as the entrainment probability, p_{ent}, as defined by Eq. (S23).

d) The carbon in the char exiting the fuel reactor, $F_{\mathrm{C,FRout}}$, must be equal to the ungasified carbon from the char in the fuel reactor.

$$F_{FRout,C} = f_{C,fx}F_{in,C} - \int_0^{H_b}(1-\delta_b)\Big[\big(-\bar{r}_{g,H_2O}\big)_{char} + \big(-\bar{r}_{g,CO_2}\big)_{char}\Big]_e S_{\mathrm{react}}\mathrm{d}z - \int_{H_b}^{H_r}\xi_{\text{g-s}}\Big[\big(-\bar{r}_{g,H_2O}\big)_{char} + \big(-\bar{r}_{g,CO_2}\big)_{char}\Big]_{tr} S_{\mathrm{react}}\mathrm{d}z \quad \text{(S53)}$$

The carbon concentration in the fuel reactor must be determined to fulfil the mass balance to carbon. This is used as convergence criteria. Thus, for low values of char concentration, the sum of carbon converted and unconverted is lower than the inlet carbon. But as higher is the char concentration, higher is the carbon gasified in the reactor. Then, there is a solution for the char concentration in which the sum of carbon converted and unconverted is equal to the inlet carbon. Eventually, the carbon conversion is defined as

$$X_C = \frac{F_{in,C} - F_{FRout,C}}{F_{in,C}} \quad \text{(S54)}$$

2.5. Calculation procedure. To solve the mathematical model developed with the above equations, convergence of the mass balance for solids and gases must occur simultaneously. The model was solved using a Visual Fortran© code. To obtain the variation of the gases concentration with the height, the entire reactor was divided into compartments with a height of Δz. The flow diagram for the overall solution of the model is shown in Figure S2.

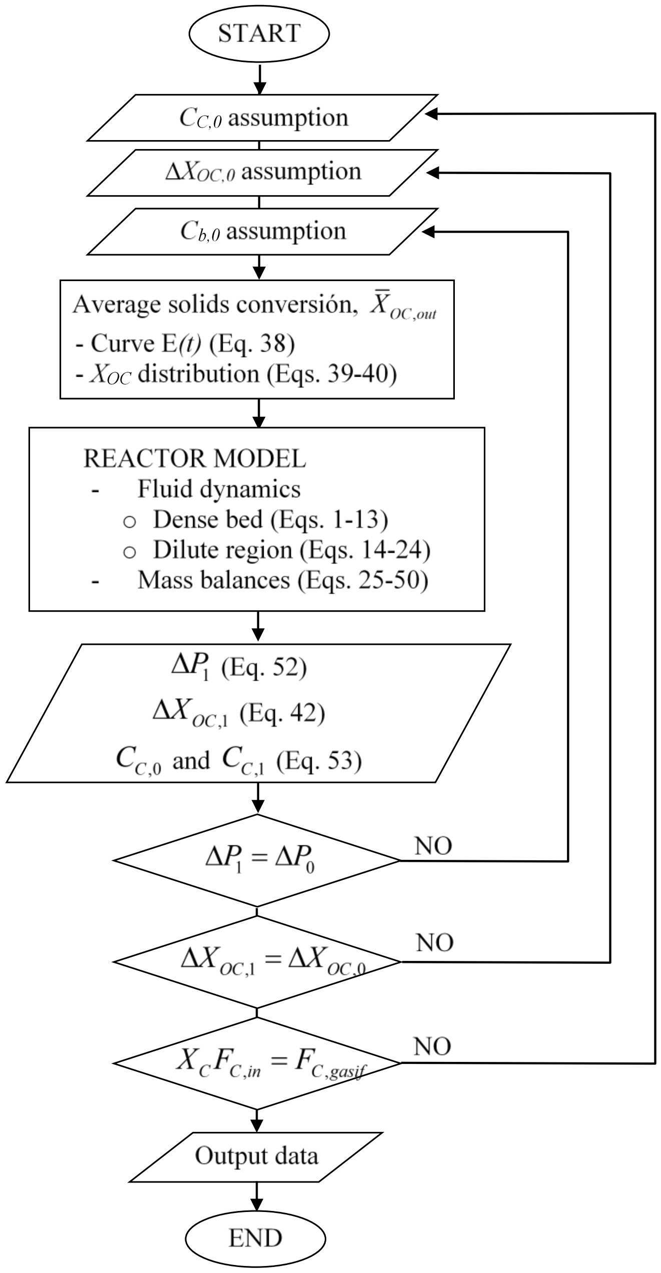

Figure S2. Simplified flow diagram of calculation for the overall solution.

The algorithm for the reactor model has the following calculation flow structure:

- Input data: the input data are the operating conditions and the characteristics of the reactor, oxygen carrier and fuel, as it is shown in Tables S1-S4.
- Firstly, it is necessary to assign a value for the following parameters: (1) solids concentration

in the dense bed, to be used in Eq. (S52); (2) average variation of the solids conversion in the reactor, $\Delta X_{OC,0} = M_O \Omega_{fuel} F_{fuel} / F_{OC} R_{OC}$, i.e. λ_{FR}=1 in Eq. (S42); and (3) carbon concentration in the dense bed, $C_{C,0} = M_C F_{C,in}/F_{OC}$.

- The distribution of conversion for the oxygen carrier in the reactor is calculated from Eqs. (39) and (40) to obtain $\Delta X_{OC} = \overline{X}_{OC,out} - \overline{X}_{OC,in}$.

- Fuel conversion: the mass balance for each reacting gas (CH_4, CO, H_2, CO_2 and H_2O) is calculated in every segment for the emulsion and bubble phases in the dense bed and the dilute region, see Eqs. (S25), (S26) and (S28). Thus, the reaction rate of the gaseous compounds (CO, H_2 and CH_4) with the oxygen carrier and the gasification rate of carbon in char by H_2O and CO_2 are calculated in each compartment. The fluid dynamical characteristics of the reactor (solids concentration and gas flow) are calculated simultaneously with the mass balance. The system of differential equations for the mass balance in every compartment was solved using a Runge–Kutta method over the entire reactor and starting from the distributor plate.

- Output data: when the mass balance for all the species is solved, the concentration profiles of the gas species are obtained along the entire reactor, as well as the variation with the height of fluid dynamical properties, e.g. gas flow distribution and solids concentration. Eventually, the concentration of the different gases and solids conversion at the reactor exit are determined. From these results, the combustion efficiency and the corresponding variation of the solids conversion, $\Delta X_{OC,1}$, is obtained from Eq. (S42). Also the carbon gasified in the whole reactor is calculated from Eq. (S53).

- Convergence of pressure drop: the pressure drop in the fuel reactor is calculated, ΔP_1, from the solids concentration profile; see Eq. (S52). This value is compared to the initial value ΔP_0.

If $\Delta P_1 = \Delta P_0$, the assumed solids concentration of the dense bed is right. If $\Delta P_1 \neq \Delta P_0$, a new value of C_b is considered; see Eqs. (S12) and (S13).

- Convergence of oxygen balance: the variation of the solids conversion, $\Delta X_{OC,1}$, obtained with the model is compared to the assumed value. If the calculated value for $\Delta X_{OC,1}$ is not the same as the input value, $\Delta X_{OC,0}$, the mass balance is repeated using a new $\Delta X_{OC,0}$ value until the two values agree. The solids flow to the cyclone is equal to the solids circulation rate in Table S2. This procedure allows to calculate the entrainment probability of oxygen carrier particles to the cyclone.

- Convergence of carbon balance: the calculated carbon in gases in the whole reactor is compared to the converted carbon in the CLG unit, i.e. carbon conversion in Table S2. If both values are not equal, a new value for $C_{C,0}$ is assumed. This procedure allows to calculate the entrainment probability of char particles to the cyclone.

- Once the pressure balance and the mass balances for oxygen and carbon have converged, the final results are obtained.

ACKNOWLEDGMENT

This work was supported by the European Union's Horizon 2020 – Research and Innovation Framework Programme under grant agreement No 817841 (Chemical Looping gsification foR sustainAble production of biofuels – CLARA). The authors thank the funding received from Gobierno de Aragon through the Combustion and Gasification project (T05_23R).

2. NOMENCLATURE

A_0 = area of the gas-distributor per nozzle, m^2 per nozzle

A_c = cross section area of core, m^2

Ar_i = Archimedes number of particle i

b = stoichiometric coefficient

C_b = concentration of solids in the dense bed, kg m^{-3}

$C_{b,i}$ = concentration of gas i in the bubble phase, mol m^{-3}

C_C = char concentration, kg of char per kg of solids

C_{dil} = concentration of solids in the dilute region, kg m^{-3}

$C_{dil,i}$ = concentration of gas i in the dilute region, mol m^{-3}

$C_{e,i}$ = concentration of gas i in the emulsion phase, mol m^{-3}

$C_{p,i}$ = concentration of gas i in the particle, mol m^{-3}

$C_{ps,i}$ = concentration of gas i at the particle's surface, mol m^{-3}

C_{tr} = concentration of solids in the transport phase, kg m^{-3}

C_z = gas concentration in the bulk gas at the height z, mol/m^3

d_b = diameter of bubble, m

d_{bottom} = inside diameter of the bottom reactor, m

d_f = average diameter of fuel particles, m

d_{oc} = average diameter of oxygen carrier particles, m

d_{up} = inside diameter of the upper part of the reactor, m

D_g = gas diffusivity, m^2/s

$E(t)$ = residence time distribution curve

$E_{a,r}$ = activation energy of oxygen carrier reduction, kJ/mol

$E_{a,1}$ = activation energy of the kinetic constant for the char gasification, kJ/mol

$E_{a,2}$ = activation energy of the adsorption constant of gasifying agent for the char gasification, kJ/mol

$E_{a,3}$ = activation energy of the adsorption constant of gasification product for the char gasification, kJ/mol

f_b = empirical function given by Eq. (S7)

$f_{C,fx}$ = fraction of fixed carbon in coal

$F_{0,i}$ = flow rate of solids entrained from the dense bed for particle i, kg/s

$F_{b,i}$ = flow of gas i in the bubble phase, mol/s

F_c = net solids flow by the core, kg/s

$F_{FRout,C}$ = flow of carbon exiting the fuel reactor, mol/s

$F_{dil,i}$ = flow of gas *i* in the dilute phase, mol/s

$F_{e,i}$ = flow of gas *i* in the emulsion phase, mol/s

F_{exc} = excess of flow in the emulsion over the minimum fluidization condition, mol/s

F_{fuel} = rate of fuel feeding, kg/s

$F_{g,CS}$ = gas flow from the carbon stripper, m^3/s

$F_{g,fuel}$ = gas flow by the fuel feeding point, m^3/s

$F_{g,in}$ = inlet gas flow, m^3/s

$F_{g,LS}$ = gas flow from the loop seal, m^3/s

F_i = molar flow of gas *i*, mol/s

$F_{in,i}$ = inlet molar flow of *i*, mol/s

F_{OC} = oxygen carrier circulation rate, kg/s

F_s = solids circulation rate or solids flow going to cyclone, kg/s

$F_{vol,i}$ = flow of gas *i* coming from volatiles, mol/s

F_w = solids flow by the wall–layer, kg/s

$F_{WGS,i}$ = flow of gas *i* due to the WGS reaction, mol/s

g = acceleration due to gravity, $m^2\ s^{-1}$

H_b = upper dense bed height, m

H_r = height of the reactor, m

$k_{0,r}$ = preexponential factor of the kinetic constant for the oxygen carrier reduction, $mol^{1-n}\ m^{3n-2}\ s^{-1}$

$k_{0,1}$ = preexponential factor of the kinetic constant for the char gasification, $s^{-1}\ atm^{-1}$

$k_{0,2}$ = preexponential factor of the adsorption constant of gasifying agent for the char gasification, atm^{-1}

$k_{0,3}$ = preexponential factor of the adsorption constant of gasification product for the char gasification, atm^{-1}

k_b = backflow ratio

k_{be} = mass transfer coefficient between bubble and emulsion, s^{-1}

k_g = external gas mass transfer coefficient, s^{-1}

K_i = decay factor for the solids concentration of *i* in the transport phase

n = reaction order

m_C = mass of carbon in char particles, kg

M_C = atomic mass of carbon, kg/mol

M_O = atomic mass of oxygen, kg/mol

n = reaction order for the conversion of the solid

p_{ent} = entrainment probability

P_i = partial pressure of gas i, atm

P_{th} = thermal power, kW

r_g = grain radius, m

$\left(-\overline{r}_{g,i}\right)_{OC}$ = average reaction rate of gas i with the oxygen–carrier, mol m^{-3} s^{-1}

$\left(-\overline{r}_{g,i}\right)_{char}$ = average reaction rate of gas i with char, mol m^{-3} s^{-1}

$\left(-\overline{r}_{OC,i}\right)$ = average reaction of oxygen in the oxygen–carrier, mol m^{-3} s^{-1}

r_p = particle radius, m

R_g = constant of ideal gases, J mol^{-1} K^{-1}

R_{OC} = oxygen transport capacity of the oxygen carrier

$\mathrm{Re}_{p,i}$ = Reynolds number for the particle i

$\mathrm{Re}_{p,mf}$ = Reynolds number for a particle at minimum fluidization velocity

S_{react} = cross section area of the reactor, m^2

Sc = Schmidt number

Sh = Sherwood number

t = time, s

t_{mr} = mean residence time of solids in the reactor, s

t_r = reacting time of solid from zero conversion until the maximum variation in solid conversion, s

T = temperature, K

T_{FR} = temperature of the fuel reactor, ºC

$u_{b\infty}$ = velocity of a single bubble, m s^{-1}

$u_{s,c}$ = velocity of solids in the core, m s^{-1}

u_g = gas velocity in the reactor, m/s

u_{mf} = gas velocity at minimum fluidization condition, m/s

u_t = terminal velocity of particles, m/s

u_{tf} = throughflow of gas in the dense bed, m/s

u_{vis} = visible gas flow in the bubbles, m/s

V = volume, m^3

X_C = conversion of carbon in char

$X_{C,FR}$ = total carbon conversion in the fuel reactor

X_{OC} = conversion of the oxygen carrier

$\bar{X}_{OC,in}$ = average conversion of the oxygen–carrier at the fuel–reactor inlet

$\bar{X}_{OC,out}$ = average conversion of the oxygen–carrier at the fuel–reactor outlet

X_s = solids conversion

$y_{e,i}$ = molar fraction of gas i in the emulsion

z = height or vertical position in the reactor, m

Greek symbols:

δ_b = fraction of bubbles in the dense bed

δ_w = thickness of the annulus, m

$\delta_{w,sat}$ = thickness of the annulus at saturation conditions, m

ΔP_0 = pressure drop in the reactor, Pa

ΔP_b = pressure drop in the bottom bed, Pa

ΔP_d = pressure drop in the dilute region, Pa

ΔX_{OC} = variation of the conversion of oxygen–carrier in the reactor

ε_b = bed porosity

$\varepsilon_{b,sat}$ = bed porosity at saturation condition

ε_{mf} = porosity at minimum fluidization condition

ε_z = porosity in the dilute region

φ_f = sphericity of fuel particles

φ_{oc} = sphericity of oxygen carrier particles

λ_{FR} = oxygen to fuel ratio in the fuel reactor

μ_g = viscosity of gas, kg m^{-1} s^{-1}

ρ_g = gas density, kg/m^3

ρ_f = apparent density of fuel particles, kg/m^3

ρ_{oc} = apparent density of oxygen carrier particles, kg/m^3

ρ_m = molar density, mol/m^3

ρ_s = average density of solids, kg/m^3

ξ_{g-s} = efficiency of contact between gas and solids in the freeboard

τ_i = time for complete solid conversion, s

τ_m = time for complete conversion of solid at the average gas concentration in the reactor, s

Ω_{fuel} = oxygen demand of the fuel, mol of oxygen per kg of coal

Ω_O = oxygen demand of gases

Ψ = ratio of the visible bubble flow to the total flow through the bubbles

3. REFERENCES

(S1) Condori, O.; Abad, A.; García-Labiano, F.; de Diego, L. F.; Izquierdo, M. T.; Adánez, J. Parametric evaluation of clean syngas production from pine forest residue by chemical looping gasification at the 20 kW_{th} scale. *J Cleaner Production* **2024**, 436, 140434. DOI: 10.1016/j.jclepro.2023.140434.

(S2) Condori, O.; Abad, A.; Izquierdo, M. T.; de Diego, L. F.; García-Labiano, F.; Adánez, J. Assessment of the chemical looping gasification of wheat straw pellets at the 20 kW_{th} scale. *Fuel* **2024**, 344, 128059. DOI: 10.1016/j.fuel.2023.128059.

(S3) Abad, A.; Adánez, J.; Cuadrat, A.; García-Labiano, F.; Gayán, P.; de Diego, L. F. Kinetics of redox reactions of ilmenite for chemical-looping combustion. *Chemical Engineering Science* **2011**, 66, 689-702. DOI: 10.1016/j.ces.2010.11.010.

(S4) Abad, A.; Condori, O.; de Diego, L.F.; García-Labiano, F. Determining intrinsic biomass gasification kinetics and its application on gasification of pelletized biomass: simplifying the process for use in chemical looping processes. Fire (MDPI), 2025, submitted for publication.

(S5) Matthesius, G. A.; Morris, R. M.; Desai, M. J. Prediction of the volatile matter in coal from ultimate and proximate analyses. *J. S. Afr. Inst. Min. Metall.* **1987**, 87, 157-161. https://hdl.handle.net/10520/AJA0038223X_1801.

(S6) Neves, D.; Thunman, H.; Matos, A.; Tarelho, L.; Gómez-Barea, A. Characterization and prediction of biomass pyrolysis products. *Progress in Energy and Combustion Science* **2011**, 37, 611-630. DOI: 10.1016/j.pecs.2011.01.001.

(S7) Pallerès, D.; Johnsson, F. Macroscopic modelling of fluid dynamics in large-scale circulating fluidized beds. *Prog. Energy Combust. Sci.* **2006**, 32, 539–569. DOI: 10.1016/j.pecs.2006.02.002.

(S8) Grace, J. R. Contacting modes and behaviour classification of gas—solid and other two-phase suspensions. *Canadian Journal of Chemical Engineering* **1986**, 64, 353-363.

(S9) Broadhurst, T. E.; Becker, H.A. 1975. Onset of fluidization and slugging in beds of uniform particles. *AIChE J.* **1975**, 21, 238-247.

(S10) Darton, R. C.; LaNauze, R. D.; Davidson J. F.; Harrisson, D. Bubble growth due to coalescence in fluidized beds. *TransIChemE* **1977**, 55.

(S11) Abad, A.; Gayán, P.; de Diego, L. F.; García-Labiano, F.; Adánez, J. Fuel reactor modelling in chemical-looping combustion of coal: 1. Model formulation. *Chemical Engineering Science* **2013**, 87, 277-293. DOI: 10.1016/j.ces.2012.10.006.

(S12) de Diego, L. F.; Gayán, P.; Adánez, J. 1995. Modelling of flow structure in circulating fluidized beds. *Powder Technology* **1995**, 85, 19-27.

(S13) Haider, A.; Levenspiel, O. Drag coefficient and terminal velocity of spherical and nonspherical particles. *Powder Technology* **1989**, 58, 63-70.

(S14) Win, K. K.; Nowak, W.; Matsuda, H.; Hasatani, M.; Bis, Z.; Krzywanski, J.; Gakewski, W. Transport velocity of coarse particles in multi-solid fluidized bed. *J. Chem. Eng. Japan* **1995**, 28, 535-540.

(S15) Harris, A.T.; Davidson, J.F.; Thorpe, R.B. Influence of Exit Geometry in Circulating Fluidized-Bed Risers. *AIChE J.* **2003**, 49, 52-64.

(S16) Johnsson, F.; Vrager, A.; Tikma, T.; Leckner, B. Solids flow pattern in the exit region of a CFB-furnace. Influence of geometry. *Proceedings of the 15th International Conference on Fluidized Bed Combustion*, Savannah, USA, **1999**.

(S17) Mohn, P.; Ströhle, J.; Epple, B. Enhancing Solid Circulation in Chemical Looping Processes Based on Cold Flow Model Tests. *Proceedings of the 25th International Conference on Fluidized Bed Combustion*, Nanjing, China, **2025**, paper 35.

(S18) Furusaki, S.; Kikuchi, T.; Miyauchi, T. Axial Distribution of Reactivity Inside a Fluid-Bed Contactor. *AIChE J.* **1976**, 22, 354-361.

(S19) Abad, A.; Adánez, J.; de Diego, L. F.; Gayán, P.; García-Labiano, F.; Lyngfelt, A. Fuel reactor model validation: Assessment of the key parameters affecting the chemical-looping

combustion of coal. *Int J Greenhouse Gas Control* **2013**, 19, 541-551. DOI: 10.1016/j.ijggc.2013.10.020.

(S20) Dewaele, O.; Froment, G.F. TAP Study of the Mechanism and Kinetics of the Adsorption and Combustion of Methane on Ni/Al_2O_3 and NiO/Al_2O_3. *Journal of Catalysis* **1999**, 184, 499-513.

(S21) Graven, W.; Long, J. Kinetics and mechanisms of the two opposing reactions of the equilibrium $CO + H_2O \leftrightarrow CO_2 + H_2$. *J. Am. Chem. Soc.* **1954**, 76, 2602-2608.

(S22) Foka, M.; Chaouki, J.; Guy, C.; Klvana, D. Gas phase hydrodynamics of a gas-solid turbulent fluidized bed reactor. *Chemical Engineering Science* **1996**, 51, 713-723.

(S23) Adánez, J.; Cuadrat, A.; Abad, A.; Gayán, P.; de Diego, L. F.; García-Labiano, F. Ilmenite Activation during Consecutive Redox Cycles in Chemical-Looping Combustion. *Energy Fuels* **2010**, 24, 1402–1413.

(S24) Palchonok. G. PhD Thesis, Chalmers University of Technology, Göteborg, Sweden, **1998**.

(S25) Chakraborty, R.K.; Howard, J.R. Combustion of char in shallow fluidized bed combustors: influence of some design and operating parameters. *J. Inst. Energy* **1981**, 54, 48-54.

(S26) Adánez, J.; García-Labiano, F.; de Diego, L. F.; Abad, A.; Gayán, P.; Mendiara, T.; Izquierdo, M. T.; Condori, O. Reaction kinetics of oxygen carrier redox reactions. *Deliverable 3.7. in Chemical Looping Gasification for Sustainable Production of Biofuels (CLARA)*, **2022**, H2020 Research and Innovation action, Grant Agreement no 817841. https://clara-h2020.eu/deliverables

(S27) Abad, A.; Adánez, J.; García-Labiano, F.; de Diego, L.F.; Gayán, P.; Celaya, J. Mapping of the range of operational conditions for Cu-, Fe-, and Ni-based oxygen carriers in chemical-looping combustion. *Chemical Engineering Science* **2007**, 62, 533-549.
